# Measuring sequencer size bias using REcount: a novel method for highly accurate Illumina sequencing-based quantification

**DOI:** 10.1186/s13059-019-1691-6

**Published:** 2019-04-29

**Authors:** Daryl M. Gohl, Alessandro Magli, John Garbe, Aaron Becker, Darrell M. Johnson, Shea Anderson, Benjamin Auch, Bradley Billstein, Elyse Froehling, Shana L. McDevitt, Kenneth B. Beckman

**Affiliations:** 1 University of Minnesota Genomics Center, Minneapolis, MN 55455 USA; 2 Department of Genetics, Cell Biology, and Development, University of Minnesota, Minneapolis, MN 55455 USA; 3 Department of Medicine, University of Minnesota, Minneapolis, MN 55455 USA; 4 Stem Cell Institute, University of Minnesota, Minneapolis, MN 55455 USA; 5 Vincent J. Coates Genomics Sequencing Laboratory, University of California, Berkeley, CA 94720 USA; 6 Present Address: Illumina, Inc, San Diego, CA 92122 USA

**Keywords:** Next-generation sequencing, DNA library preparation, PCR-free, Illumina, Size bias, RNA-Seq, RAD-Seq, Genotyping by sequencing, ATAC-Seq

## Abstract

Quantification of DNA sequence tags from engineered constructs such as plasmids, transposons, or other transgenes underlies many functional genomics measurements. Typically, such measurements rely on PCR followed by next-generation sequencing. However, PCR amplification can introduce significant quantitative error. We describe REcount, a novel PCR-free direct counting method. Comparing measurements of defined plasmid pools to droplet digital PCR data demonstrates that REcount is highly accurate and reproducible. We use REcount to provide new insights into clustering biases due to molecule length across different Illumina sequencers and illustrate the impacts on interpretation of next-generation sequencing data and the economics of data generation.

**Electronic supplementary material:**

The online version of this article (10.1186/s13059-019-1691-6) contains supplementary material, which is available to authorized users.

## Background

Engineered constructs underlie many experimental techniques in genetics and genomics. For example, targeted perturbation of gene function using RNA interference or CRISPR/Cas9 allows for pooled genome-wide genetic screens that can be read-out through next-generation sequencing (NGS) of the small hairpin RNA (shRNA) [1, 2] or synthetic guide RNA (sgRNA) [3–6] constructs, or associated sequence tags/barcodes [7]. Transposable elements are also commonly used to mutate or otherwise manipulate genetic loci, and similarly enable genome-scale saturation mutagenesis screens in which the transposon-genome junction is measured using NGS [8]. Lineage tracing [9, 10] and connectomics [11, 12] approaches also rely on NGS-based quantification of molecular tags. In all of these approaches, polymerase chain reaction (PCR) amplification is used to enrich for the sequence tags and to add adapters and other functionalities (e.g., sample-specific barcodes) required for sequencing. However, PCR introduces bias into these measurements. Sequence tags comprised of shRNAs, sgRNAs, transposon-genome junctions, or synthetic barcodes can all differ in primary sequence and biophysical properties, which, along with other variables such as template concentration and PCR conditions, can influence amplification efficiency in unpredictable ways [13–15]. Adding unique molecular identifiers (UMIs) can mitigate some of this bias, but increases the complexity of both library preparation and analysis [16, 17]. Other approaches such as droplet digital PCR (ddPCR) and NanoString analysis can be used to overcome the quantitative inaccuracies associated with measuring engineered genetic constructs [18, 19]. The NanoString nCounter instrument uses hybridization of fluorescently barcoded probes to count copies of target molecules in a sample. ddPCR achieves high accuracy by partitioning individual molecules into emulsion droplets and counting the number of droplets with and without amplification, thereby digitizing PCR and removing amplification bias from the quantification process. However, ddPCR and NanoString analysis, while highly accurate, lack the throughput and resolution afforded by NGS.

**Fig. 1:**
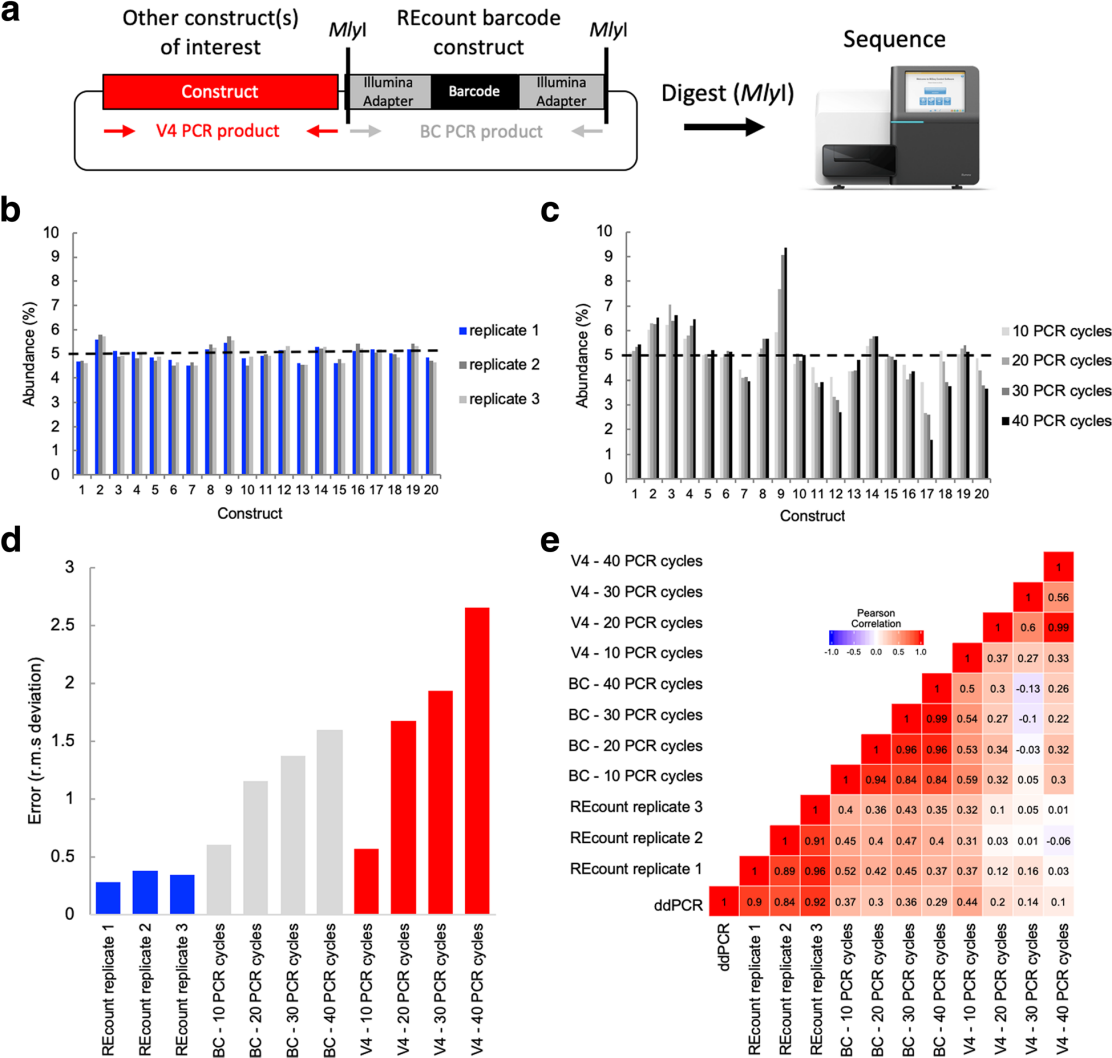
REcount enables accurate and precise measurements of plasmid pools. **a** Design of REcount constructs. A barcode-containing, Illumina adapter-flanked construct is liberated with a restriction enzyme (*Mly*I) digest and directly sequenced. **b** Accuracy and reproducibility of REcount. **c** Analogous measurements of the same plasmid pool shown in panel **b** using varying PCR cycle numbers. **d** Root mean squared deviation from expected values (5% per construct) when the plasmid pool is measured using REcount, and varying cycles of PCR amplification of either the barcode construct (BC) or another variable sequence in these plasmids (V4). **e** Pearson correlation heatmap comparing REcount measurements with droplet digital PCR data and with conventional PCR amplification of either the BC or V4 amplicons

We have developed a novel method, REcount (*R*estriction *E*nzyme enabled *count*ing) for quantifying sequence tags associated with engineered constructs that is straightforward to implement and allows for direct NGS-based counting of a potentially enormous number of sequence tags. In this approach, an Illumina adapter-flanked DNA barcode is liberated by digesting with *Mly*I (a type IIS restriction enzyme that produces blunt-ended molecules) and sequenced to directly count template molecule abundance (Fig. 1a). We demonstrate that REcount measurements are amenable to multiplexing through the use of five orthogonal restriction enzymes, an approach that likely is further generalizable to other enzymes.

We used REcount to design a set of synthetic DNA standards that can be used to assess clustering bias due to molecule length on Illumina sequencers, and demonstrate that there is substantial variation in size bias between different Illumina instruments. Specifically, molecules in DNA sequencing libraries are systematically and often substantially over- or under-represented on different Illumina sequencer models in a manner related to molecule length. Finally, we assess the impact of size bias across several common applications of NGS, including transcriptomic measurements (RNA-Seq [20]), reduced-representation genotyping (RAD-Seq/GBS [21]), and accessible chromatin profiling (ATAC-Seq [22]).

## Results

### Development and assessment of the REcount method

In order to characterize the REcount method, we constructed a pool of 20 synthetic plasmids containing REcount barcodes, mixed at an equimolar abundance (5% per plasmid) based on fluorometric DNA concentration measurements. This pool was digested with *Mly*I and sequenced on an Illumina MiSeq. All 20 barcodes were detected at relative abundances ranging from 3.41 to 6.32% (CV = 0.13), consistent with the targeted abundances of 5% per construct (Additional file 1: Figure S1). To generate a more accurately pooled reference standard for subsequent experiments, we used this sequencing data as the basis for re-pooling the 20 plasmids and digested the new pool with *Mly*I and sequenced. The range of relative abundances of the re-pooled plasmids was narrower, ranging from 4.52 to 5.58% (CV = 0.06), indicating that the initial sequencing data was predictive in improving the accuracy of pooling as assessed by REcount (Additional file 1: Figure S1). To assess the reproducibility of these measurements, we digested and sequenced two additional replicates of the even plasmid pool. The replicate REcount measurements were highly reproducible with an average CV of 0.02 (Fig. 1b).

Next, we compared REcount measurements of the even plasmid pool to PCR-based measurements, either of the barcode construct (BC) or another construct-specific sequence (V4). We tested amplification using 10, 20, 30, or 40 PCR cycles. While 40 cycles of PCR is more than would typically be used for NGS library preparation, we chose these conditions to bracket the range of low and high PCR cycle numbers. PCR-based measurements exhibited substantial construct-specific deviations from the expected 5% values, the extent of which increased with greater numbers of PCR cycles (Fig. 1c, d). Furthermore, the construct-specific deviations from expected values were uncorrelated for the BC and V4 amplicon measurements, suggesting that the PCR biases were a function of template sequence (Additional file 1: Figure S2).

ddPCR is a highly accurate method for measuring the copy number of molecules in a sample [19]. In order to independently measure the relative template concentrations in the even plasmid pool, we designed a pair of ddPCR assays targeting each barcode construct and validated the specificity of each assay using qPCR on each of the 20 individual plasmid templates (Additional file 1: Figure S3) [19]. The ddPCR-based measurements correlated well with the REcount measurements, both for the original and re-pooled even plasmid pools (Fig. 1e, Additional file 1: Figure S3). In contrast, the PCR-based measurements of both the BC and V4 amplicons were not well-correlated with the ddPCR measurements (Fig. 1e, Additional file 1: Figure S3). These results were corroborated with similar measurements of a pool of the same 20 plasmids mixed in a staggered manner, where PCR-based measurements had reduced correlation with ddPCR measurements and led to a systematic overestimation of the lower abundance constructs (Additional file 1: Figure S4). Taken together, these results indicate that REcount accurately reports on template abundance, while PCR-based measurements introduce increasing error with increased cycle numbers.

### Multiplexing REcount measurements through the use of orthogonal restriction enzymes

**Fig. 2:**
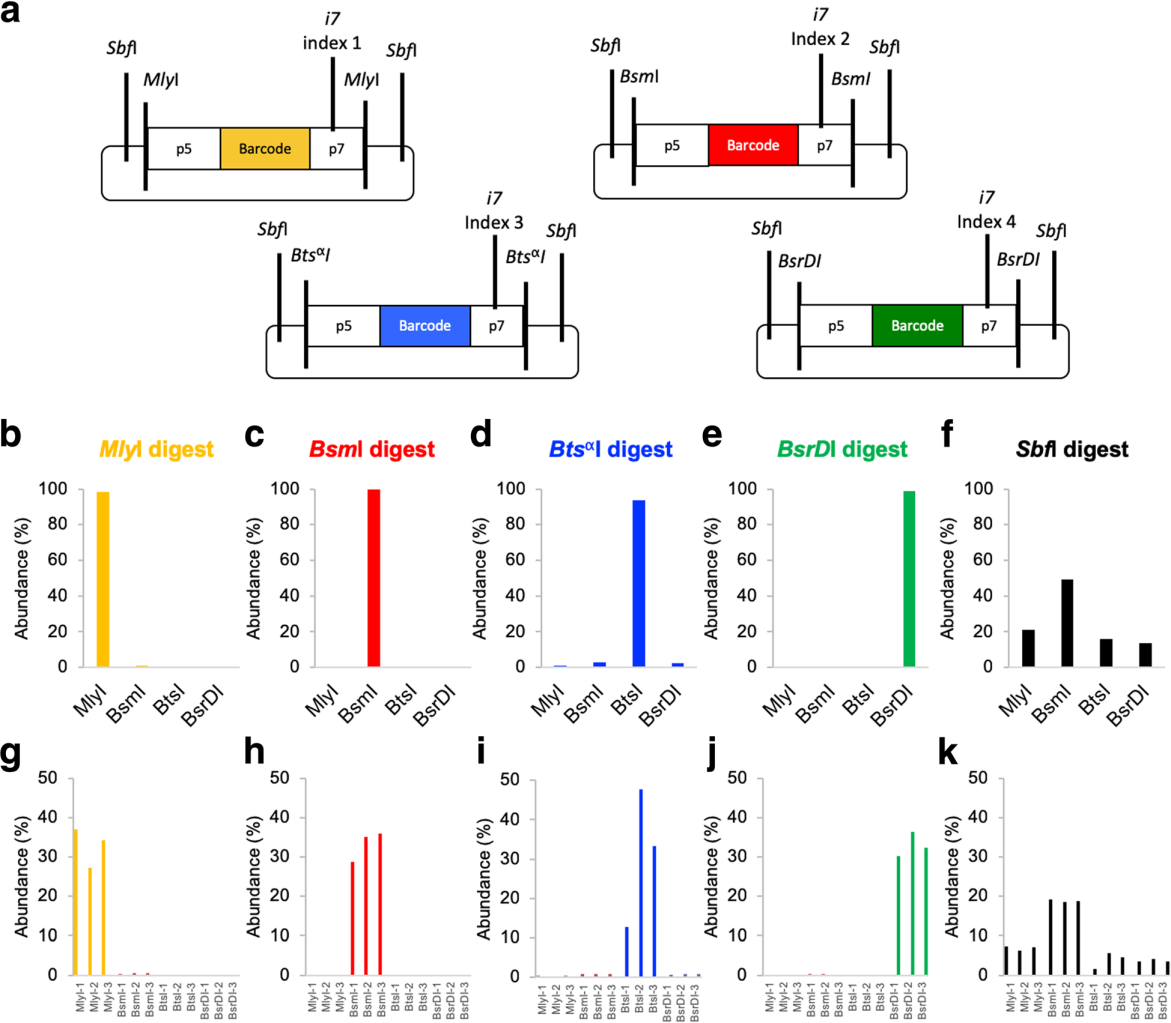
Multiplexing of REcount measurements using orthogonal restriction enzymes. **a** Plasmids containing REcount constructs flanked by orthogonal restriction enzyme cut sites. **b**–**f** Total mapped reads identified for each construct type when the plasmid pool is digested with the indicated enzyme. **g**–**k** Mapped reads identified for each construct when the plasmid pool is digested with the indicated enzyme

One drawback of the REcount method is that the indices that specify sample identity in multiplexed sequencing, which are typically flexibly added by PCR, are hard-coded into the constructs. To overcome this limitation, we tested whether orthogonal restriction enzymes could be used to multiplex REcount measurements. We initially chose *Mly*I as the flanking enzyme because it could precisely liberate the desired Illumina adapter-flanked construct. We tested whether other restriction enzymes that do not cleanly liberate flush Illumina adapter ends could also be used for REcount measurements. Initially, we tested *Bsm*I, *Bts*^α^I, and *Bsr*DI, each of which leaves 2-nt 3′ overhangs. We constructed a pool of 12 plasmids comprised of sets of three barcoded constructs flanked by either *Mly*I, *Bsm*I, *Bts*^α^I, or *Bsr*DI (Fig. 2a). In addition, all 12 of these constructs contained a pair of *Sbf*I sites located such that digestion with *Sbf*I liberates all 12 Illumina adapter-flanked cassettes with additional overhangs of between 30 and 36 bp upstream of the p5 flowcell adapter and between 40 and 50 bp downstream of the p7 flowcell adapter. We digested this plasmid pool with each of the five enzymes individually and individually sequenced the digests and mapped the reads to a reference file containing all 12 expected barcodes. For *Mly*I, *Bsm*I, *Bts*^α^I, and *Bsr*DI, the expected barcodes were detected for each respective enzyme (Fig. 2b–e, g–j). All 12 barcodes were detected when the pool was digested with *Sbf*I, indicating that clustering and sequencing can occur even in the presence of large (30–50 bp) overhangs (Fig. 2f, k). We were not able to determine whether the length of the overhang affects the efficiency of clustering as each of these samples was sequenced in a portion of a MiSeq lane, together with other libraries. We observed differing amounts of off-target barcode detection in these orthogonal digests, ranging from < 0.2% in the *Bsm*I digest to approximately 6% in the *Bts*^α^I digest (Fig. 2b–e, g–j). This could likely be improved by adding a size selection step.

### Using REcount-based size standards to measure size bias in Illumina sequencing

**Fig. 3:**
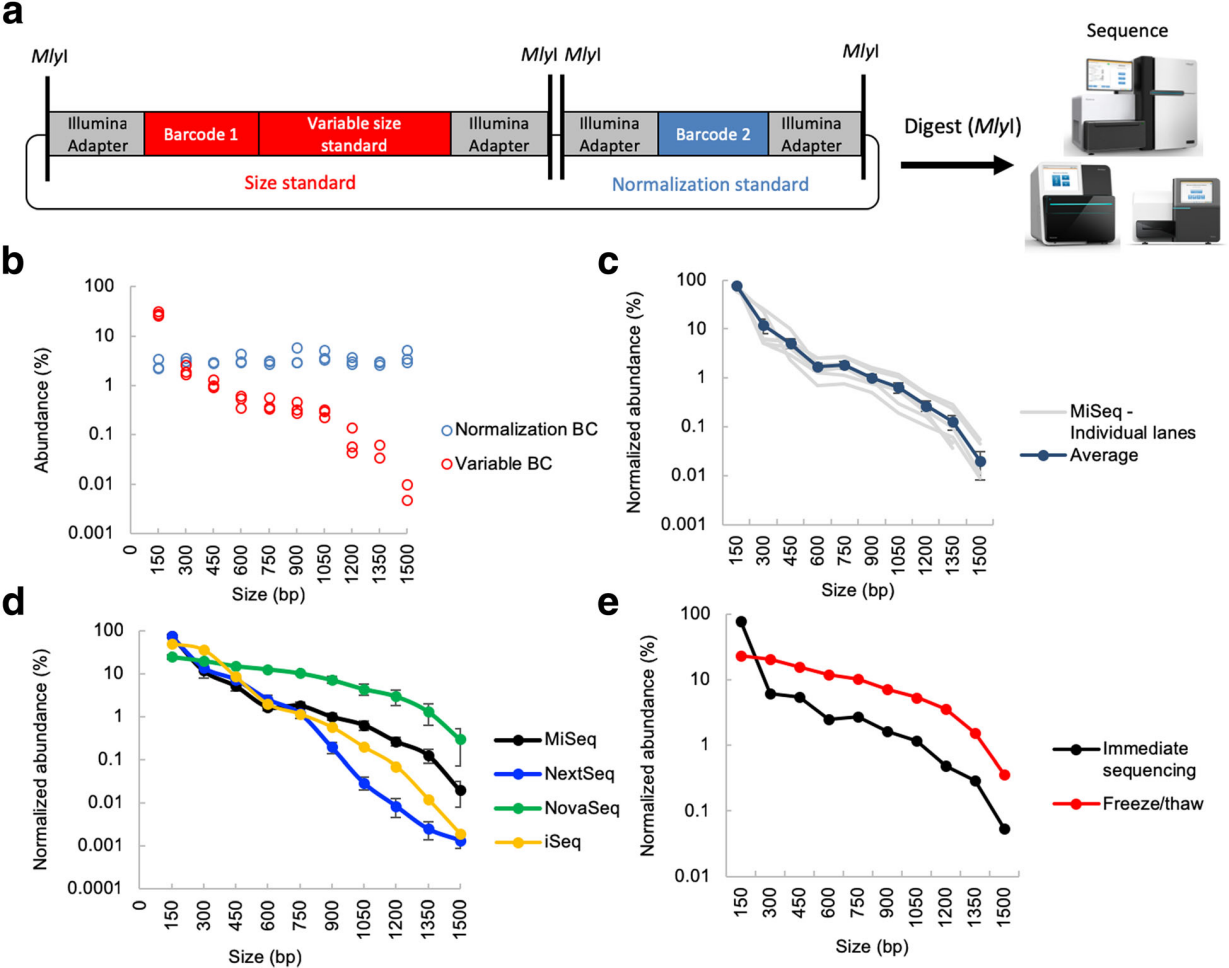
Illumina size standards allow measurement of sequencer-specific size biases. **a** Design of REcount-based Illumina size standard constructs. Each standard construct contains a normalization barcode, as well as a barcode associated with a variable size standard that can be liberated by *Mly*I digestion and directly sequenced. **b** Raw abundance data for all 30 size standards and normalization barcodes from a MiSeq run. **c** Run-to-run variability of multiple MiSeq runs (*n* = 6 flow cells). **d** Size bias profiles of the iSeq (*n* = 1 flow cell), MiSeq (*n* = 6 flow cells), NextSeq (*n* = 4 flow cells), and NovaSeq (*n* = 4 flow cells, 4 lanes) sequencers. Note: Size bias data for other Illumina instruments is shown in Additional file 1: Figure S5. **e** Size bias profiles of the same library either clustered on the MiSeq immediately after denaturation or clustered after freezing and thawing the denatured library. Error bars are ± s.e.m

While it is known that molecule length affects clustering and sequencing efficiency on Illumina sequencers [23], the extent of this bias and the degree to which it differs between different Illumina instruments has not been characterized in detail. Thus, we used REcount to characterize the size bias profiles of the Illumina iSeq, MiSeq, HiSeq 2500, HiSeq 4000, NextSeq, and NovaSeq sequencers. We synthesized 30 constructs, each of which contained an *Mly*I-flanked normalization barcode of consistent length (164 bp), and a barcode-containing variable-length insert ranging from 22 to 1372 bp, resulting in adapter-flanked molecules between 150 and 1500 bp (Fig. 3a, Additional file 1: Figure S5). In order to minimize sequence-specific artifacts, the variable-length inserts were chosen to have between 42 and 58% GC content and were comprised of 10 constructs each (spanning the full 150–1500-bp size range) derived from three different molecules; the *Escherichia coli* (*E. coli*) 16S rRNA gene (16S), the *Drosophila melanogaster* (*D. melanogaster*) *alpha-Tubulin84B* gene (Tubulin), and the *D. melanogaster glyceraldehyde-3-phosphate dehydrogenase 1* (GAPDH) gene (Additional file 1: Figure S5).

These Illumina size standard constructs were pooled at an equimolar ratio based on fluorometric DNA concentration measurements, digested with *Mly*I, and sequenced on different Illumina sequencers with no intervening clean-up step, to ensure that no material was lost. Representative data from a single MiSeq run is shown in Fig. 3b. Since each normalization barcode is present at an equimolar ratio to the corresponding size standard (as they are on the same plasmid), this allows any inaccuracies in plasmid pooling to be accounted for. Within a sequencing platform, clustering size bias exhibits run-to-run variation (Fig. 3c, Additional file 1: Figure S6). All six of the sequencers we tested exhibited preferential clustering of smaller fragments, consistent with previous anecdotal observations (Fig. 3d, Additional file 1: Figure S5). However, the magnitude of this effect and the shapes of the size bias curves differ substantially between the iSeq, MiSeq, HiSeq 2500, HiSeq 4000, NextSeq, and NovaSeq (Fig. 3d, Additional file 1: Figure S6). For the NextSeq and NovaSeq, the extent of the size bias between platforms ranged between three and five-fold for small or moderately sized molecules (150–600 bp) to up to more than 100-fold for molecules over 1 kb.

Differences were also seen between the HiSeq 2500 in Rapid Run (onboard clustering) and High Output (cBot clustering) modes (Fig. 3d, Additional file 1: Figure S5). In addition, we observed an effect of molecule length on sequencing quality score [24], with a general trend towards longer molecules having lower quality scores (Additional file 1: Figure S5). The magnitude of the effect of molecule length on sequence quality varied among the different instruments.

The denaturation process can also affect the size bias observed on Illumina instruments. Denatured libraries are sometimes saved for re-sequencing in the case of a run failure (although Illumina’s best practices recommend preparing freshly denatured libraries). To test whether freshly denatured libraries perform differently from frozen previously denatured libraries, we sequenced a freshly denatured library on a MiSeq, and the same denatured library 1 day later, after a freeze-thaw cycle, on a second MiSeq. The freeze-thaw cycle had a substantial effect on the size bias profile of this library; in particular, there was a dramatic reduction in the fraction of 150-bp molecules observed, resulting in a corresponding upward shift of the curve (Fig. 3e). It is likely that this shift reflects differential re-annealing of 150-bp fragments (which are in molar excess due to the presence of the large number of similarly sized normalization barcodes), or other small library molecules in the sequencing pool. This observation suggests that some of the difference in clustering size bias observed between the different platforms may be due to differences in denaturation conditions, the amount of time between loading the library and clustering, and whether the clustering process takes place in a chilled compartment (such as on the MiSeq) or not (such as the HiSeq 2500 and NextSeq). Consistent with this idea, the variation between HiSeq 2500 and HiSeq 4000 flow cells is much larger than the variation between the lanes on the same flow cell (Additional file 1: Figure S6).

It is also likely that a portion of the variability between flow cells is due to differences in the size distributions of the libraries being sequenced together with the synthetic size standards, as competition for clustering will occur between all molecules in the sequencing lane. We observed a shift in the curve corresponding to a decreased representation of the larger size standards when they were sequenced together with a library containing a significant amount of material that was smaller than 300 bp on the HiSeq 4000 (Additional file 1: Figure S6). Although the size standards were sequenced together with different libraries across the different instruments, this context-dependent clustering is not sufficient to explain the large differences we see between different instruments. For example, libraries with similar average sizes and distributions yielded dramatically different measurements of size bias on the NextSeq versus the HiSeq 4000 (Additional file 1: Figure S6).

Surprisingly, we also detected an instance of construct-specific size bias, specifically on the HiSeq 2500 platform in Rapid Run mode (Additional file 1: Figure S6). In contrast to the iSeq, MiSeq, HiSeq 2500 High Output, HiSeq 4000, NextSeq, and NovaSeq where no systematic construct-specific biases were observed, the size bias curves for the 16S, GAPDH, and alpha-Tubulin constructs separated as size increased, with 16S showing much less of a drop-off with increased molecule size. One possible explanation for this difference is that the 16S rRNA gene has a substantial secondary structure [25], which may serve to shorten the effective length of the molecule during the clustering process. This phenomenon may be due to differences in the clustering process or temperature on this platform, which may be less effective at dissociating the secondary structure of the 16S rRNA gene (https://support.illumina.com/bulletins/2016/10/considerations-when-migrating-nonillumina-libraries-between-sequencing-platforms.html). The HiSeq and MiSeq also have different recommended NaOH concentrations for denaturing libraries. It is possible that long molecules, particularly those with highly stable secondary structure, are incompletely denatured under the HiSeq denaturing conditions.

### Characterizing the effects of size bias on data generation across different Illumina sequencers

**Fig. 4:**
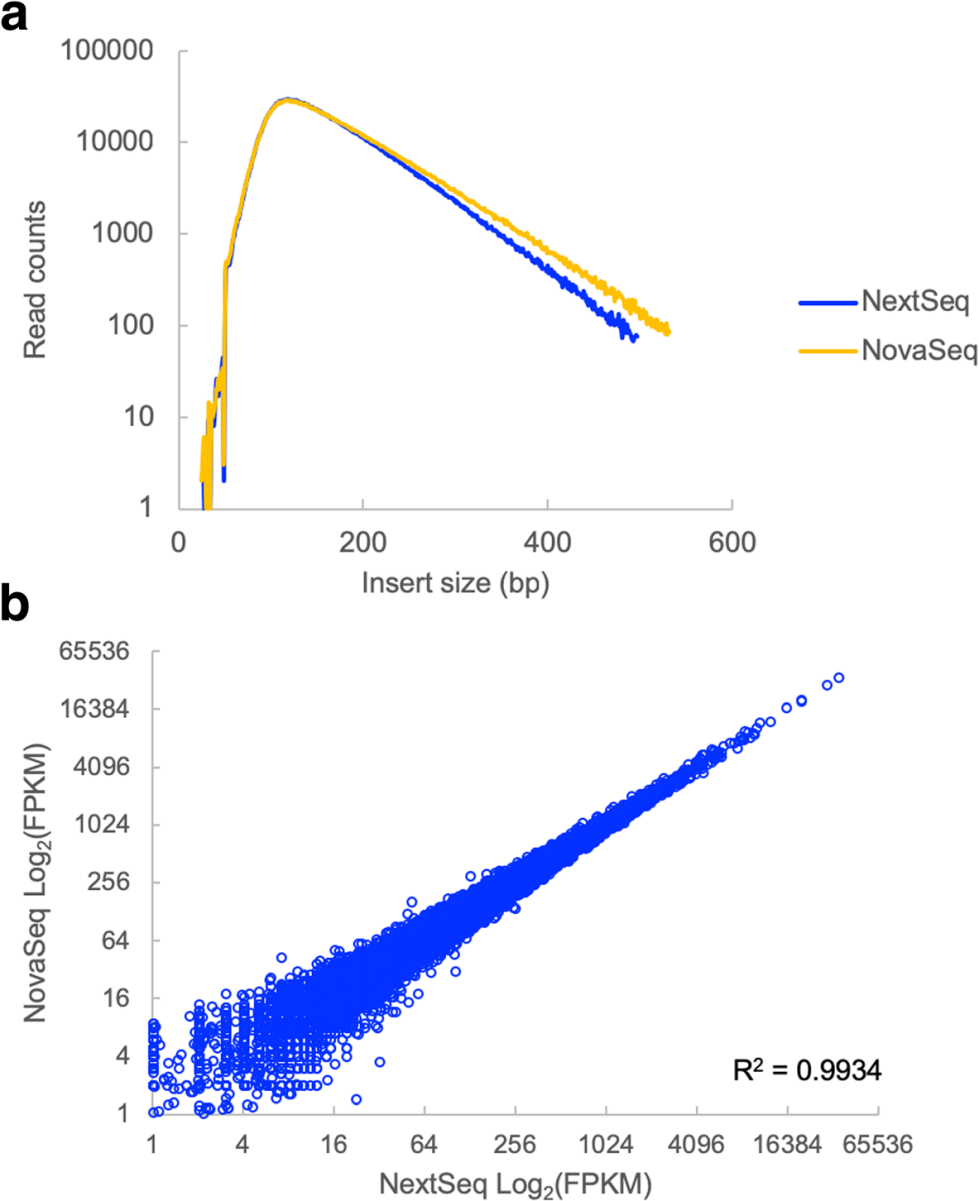
Instrument-specific size biases have minimal effect on RNA-sequencing data. **a** Fragment size distributions for an RNA-Seq library sequenced on the NovaSeq and the NextSeq. **b** Correlation of expression values (FPKM) for this library across the two instruments

In order to determine the effects of size bias on the interpretation of NGS results, we sequenced a number of different types of libraries across multiple Illumina sequencers. First, we examined an RNA-Seq library. Since RNA-Seq library preparation involves random shearing of cDNA molecules, we did not expect to see an effect of size bias on the gene expression counts. In addition, the range of fragment sizes in this library was relatively tight, resulting in only minor differences in the observed insert sizes between the NextSeq and NovaSeq platforms (Fig. 4a). Consistent with expectations, the gene expression measurements for the RNA-Seq library were highly correlated between the NextSeq and the NovaSeq (Fig. 4b).

**Fig. 5:**
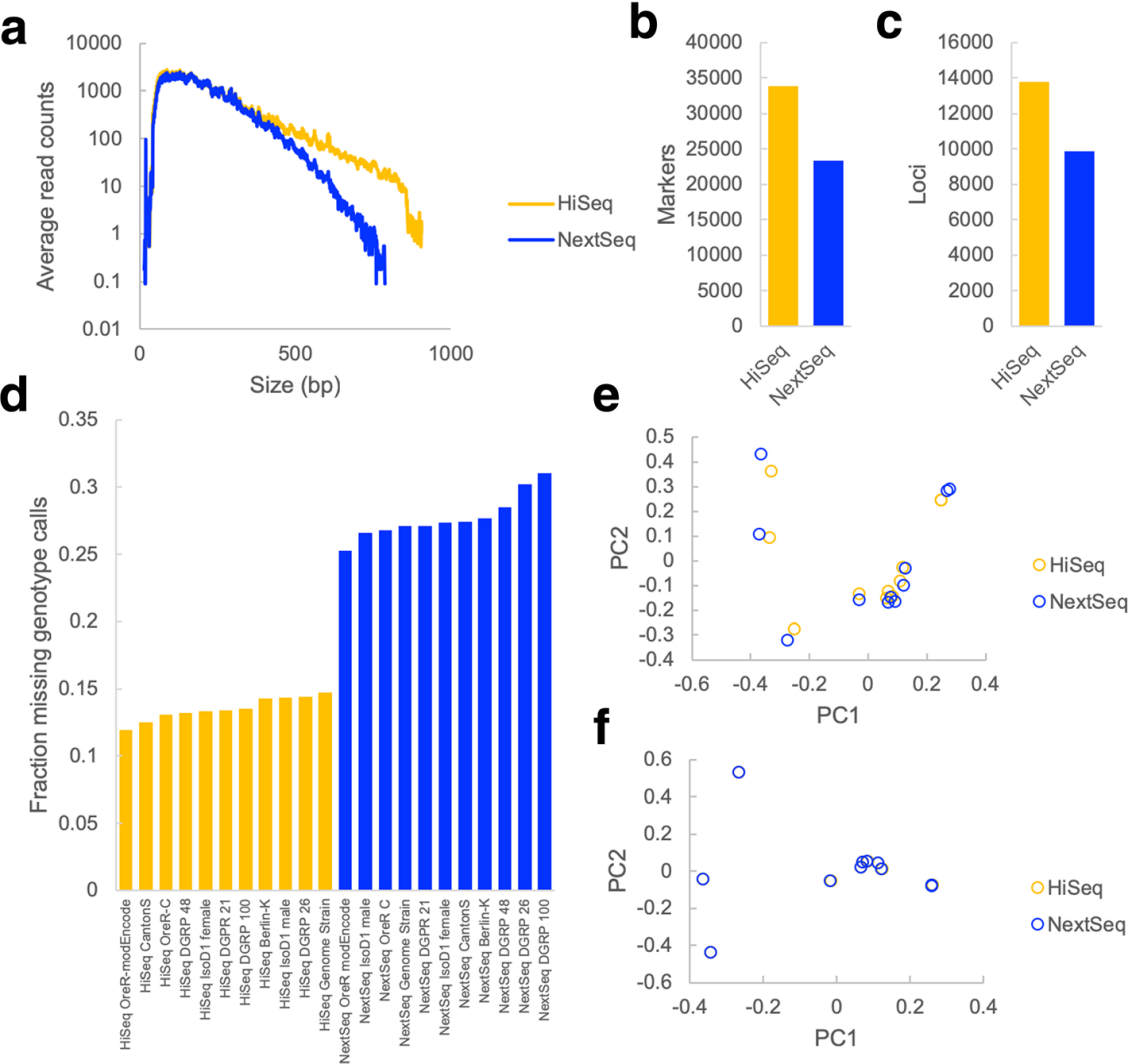
Instrument size biases affect genotyping marker observations in RAD-Seq data. **a** Average read counts for 11 RAD-Seq samples sequenced on the HiSeq or NextSeq. **b** Number of markers observed in filtered VCF file for the 11 RAD-Seq libraries. **c** Number of loci observed in filtered VCF file for the 11 RAD-Seq libraries. **d** Fraction of missing genotype calls for each sample in the unfiltered VCF file. **e** PCA plot generated using the unfiltered VCF file. **f** PCA plot using the filtered VCF file. HiSeq data points overlap with NextSeq data points in this plot

Next, we examined the effects of cross-platform size biases on two different types of libraries (RAD-Seq and ATAC-Seq) that were made using techniques which do not involve random shearing, and for which the size of fragments represented in the sequencing library are related to the underlying biology being measured. RAD-Seq (also commonly referred to as genotyping by sequencing (GBS) or sequencing-based genotyping (SBG)), is a reduced-representation genotyping method in which adapters are ligated to restriction fragments which are then sequenced to identify sequence polymorphisms in a reproducible subset of genomic loci. We made RAD-Seq libraries from 11 strains of *Drosophila melanogaster* and sequenced these libraries on both the HiSeq 2500 and the NextSeq. In contrast to the RNA-Seq library, these RAD-Seq libraries had a much wider size distribution (Additional file 1: Figure S7). As observed with the REcount size standards, the size distribution of molecules sequenced on the HiSeq 2500 skewed larger than that on the NextSeq (Fig. 5a). This resulted in a larger number of markers and loci detected on the HiSeq compared to the NextSeq when using identical read depths and filtering parameters (Fig. 5b, c). When these data were processed together, the differences in the size distribution also resulted in an elevated rate of missing genotype calls for the NextSeq samples (Fig. 5d) and a sequencer-dependent shift in the PCA plot (Fig. 5e). This shift was in general smaller than the differences between different genetic backgrounds and could be eliminated by filtering the VCF file to remove variants that were genotyped in less than 95% of the samples (Fig. 5f). Thus, in the case of RAD-Seq, size bias introduces a measurable, but correctable, bias.

The choice of platform for RAD-Seq can also affect the economics of sequencing. Since the samples sequenced on the HiSeq had a wider distribution of fragment sizes (Fig. 5a), this resulted in more markers (Fig. 5b), but a lower average coverage (Additional file 1: Figure S8). Thus, the sharper attenuation of clustering based on fragment size observed on the NextSeq can lead to more uniform coverage of a set of markers, and could potentially allow for higher levels of multiplexing. However, it should be noted that size selection could also achieve a similar effect with RAD-Seq libraries.

**Fig. 6:**
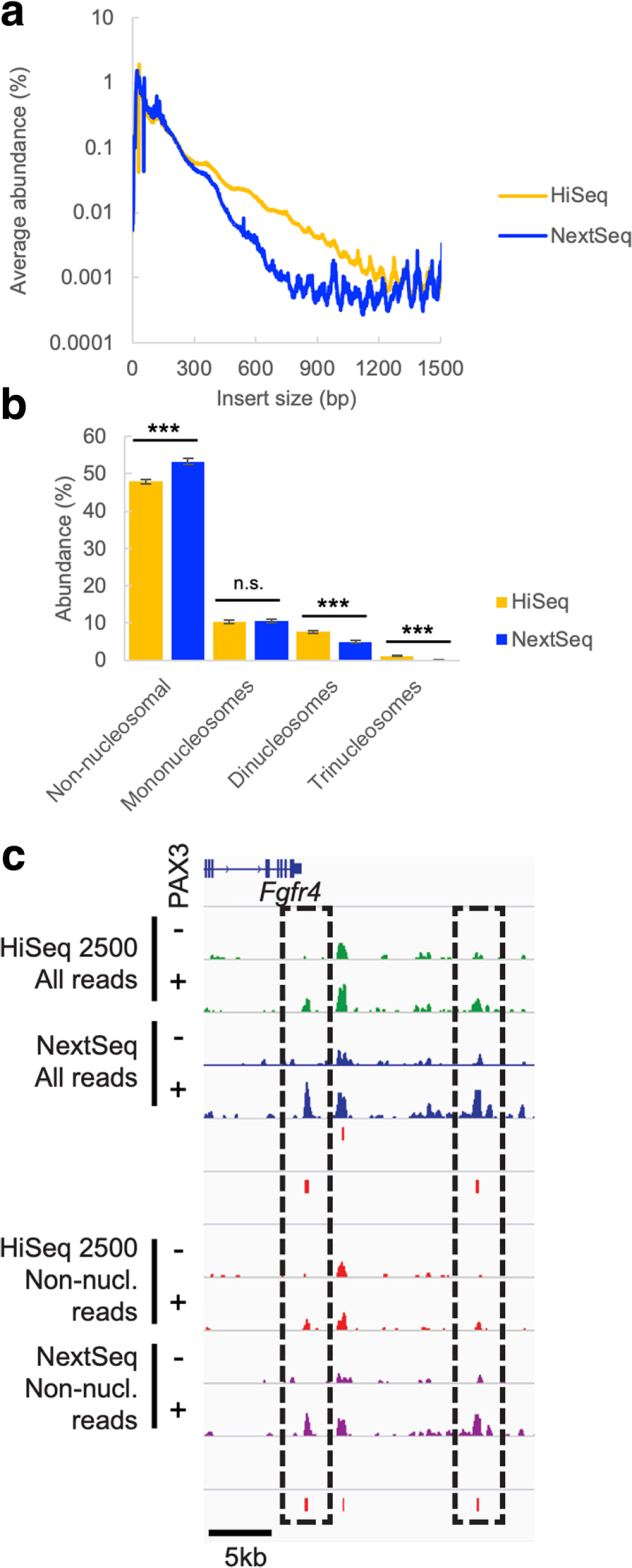
Effect of instrument size bias on ATAC-Seq data. **a** Average insert size for 6 ATAC-Seq libraries sequenced on the HiSeq or NextSeq. **b** Percentage of reads at a subsampled depth of 20 million reads per sample classified as non-, mono-, di-, and tri-nucleosomal. *n* = 6 libraries. ***denotes *p* < 0.01 using a t-test. n.s. denotes no significant difference. **c** Distribution of mapped reads at the *Fgfr4* locus. IGV plots of mapped reads for each sample, subsampled to a depth of 20 million reads, and either directly mapped (“All reads”) or split into the non-nucleosomal (“Non-nucl.”) subset and mapped. MACS peak calls for PAX3-responsive sites for HiSeq (top) and NextSeq (bottom) are below each set of mapped reads

ATAC-Seq represents another commonly used library preparation method where a wide range of DNA fragment sizes are sequenced. In this technique, library insert size is also intimately linked to the underlying biology, as the fragment sizes represent the chromatin state of the corresponding DNA. In cases such as this, size selection cannot be used to harmonize fragment sizes between different libraries, as size selecting could introduce bias into the resulting data by skewing the proportions of nucleosomal and non-nucleosomal reads. We sequenced six ATAC-Seq libraries representing three replicates each of mouse ES-derived mesodermal precursor cells (PDGFRα+FLK1− sorted cells) expressing or not expressing the PAX3 transcription factor on both the HiSeq 2500 and the NextSeq. As with the REcount size standards, larger-sized fragments were more highly represented on the HiSeq 2500 than on the NextSeq (Fig. 6a, Additional file 1: Figure S9). This resulted in a significant difference in the proportion of non-nucleosomal, di-nucleosomal, and tri-nucleosomal reads between the two sequencers, while the proportion of mono-nucleosomal reads was unaffected (Fig. 6b). It should be noted that for ATAC-Seq, reduced clustering efficiency for larger-sized fragments as well as PCR amplification bias during library preparation both likely serve to skew the representation of nucleosome-associated reads.

We examined a known PAX3-responsive enhancer at the *Fgfr4* locus [26, 27] in order to assess the effects of sequencing platform on detection of transcription factor binding sites using ATAC-Seq. Sequencing reads were mapped to the mouse genome, subsampled to a depth of 20 million reads per sample, and peaks that were enriched in PAX3-expressing cells were called using MACS [28] either for all reads or for the non-nucleosomal reads (Fig. 6c, Additional file 1: Figure S10). In both cases, at the *Fgfr4* locus, PAX3-dependent enhancer peaks were more prominent when the samples were sequenced on the NextSeq. This difference was likely due to the over-representation of smaller (non-nucleosomal) fragments on the NextSeq relative to the HiSeq 2500, which correspond to transcription factor binding sites. Using a consistent read depth and parameters, this cross-platform difference resulted in PAX3-responsive peaks being called with MACS for the NextSeq samples and not the HiSeq samples.

Thus, sequencer-specific size bias can influence biologically relevant conclusions in some instances. It is possible in this case that increased read depth on the HiSeq 2500 or alterations of the MACS peak-calling parameters could resolve this difference. Nonetheless, in cases such as ATAC-Seq, where biologically meaningful information is encoded in the size structure of the sequencing library, care should be taken to account for and minimize the effects of sequencer-specific size bias in data generation (for instance, by carrying out all experiments on the same sequencing platform, and by choosing an instrument with a desirable size bias profile).

## Discussion

In summary, we describe REcount, a novel method for obtaining highly accurate and precise PCR-free NGS-based measurements of engineered constructs. In future studies, similar constructs could be incorporated into shRNA, CRISPR, and transposon libraries to improve quantification of these molecules in pooled genetic screens. Currently, such measurements are prone to bias introduced by PCR, as we observed for both the BC and V4 amplicons (Fig. 1, Additional file 1: Figure S4), as well as other PCR artifacts such as PCR chimeras which can cause barcode-construct mis-associations [29]. Sequence-specific amplification biases are often mitigated by including input controls, which are thought to accurately model amplification biases. However, amplification biases can be impacted by template concentration and by the context of the other molecules in the amplification reaction [14] and can limit the sensitivity of these assays by compressing the dynamic range (Additional file 1: Figure S4). One challenge of employing REcount in these contexts is the large amount of genomic DNA relative to the PCR-free barcode construct. However, we have successfully quantified transposon pools from isolated *E. coli* genomic DNA using this approach (data not shown).

We further demonstrated that multiplexing of REcount measurements is possible using orthogonal restriction enzymes (Fig. 2). The fact that multiple restriction enzymes can be used to liberate REcount constructs, including *Sbf*I which leaves 30–50-bp overhangs on the Illumina adapters, strongly suggests that many other restriction enzymes could be employed for making multiplexed REcount measurements. Thus, REcount allows for potential multiplexing strategies involving orthogonal digestion of distinct subpopulations of molecules or of concatemerized barcode arrays. It is also possible that substituting recombinases for restriction enzymes could lead to more flexible barcoding strategies.

We used REcount to measure size bias on several different Illumina sequencers. We found that size bias can vary between runs and instruments and that the denaturation procedure can affect size bias (Fig. 3). Due to the competitive clustering of molecules of different sizes, it is likely that a portion of the variability between runs and lanes is due to differences in the size distributions of the libraries being sequenced together with the synthetic size standards. Such context-specific effects may be more prominent on patterned flow cell instruments, where library molecules compete for a defined number of clustering sites. Thus, the shape of the size bias curve is likely sensitive to both the size distribution of the libraries being sequenced along with the size standards, as well as the proportion of the lane devoted to the size standards.

As expected, we did not see effects of sequencer-specific size biases on randomly sheared RNA-Seq libraries (Fig. 4). In instances where library fragment sizes were generated in a non-random manner (RAD-Seq and ATAC-Seq libraries), we did see differences in the data generated on instruments with different size bias profiles. In the case of the RAD-Seq libraries, the instrument-specific signal could be removed by filtering out markers which did not appear in both sets of samples (Fig. 5). However, the choice of instrument influenced the evenness of marker coverage and thus can affect the economics of sequencing. In the case of ATAC-Seq, differences in size bias between the HiSeq 2500 and the NextSeq led to differences in the proportions of nucleosomal and non-nucleosomal reads and differences in peak calling for a known PAX3-responsive enhancer element (Fig. 6).

In sum, these results indicate that care should be taken when interpreting quantitative measurements or comparing data across different platforms. This is particularly true in cases where library size distributions are non-random such as in several chromatin profiling methods (e.g., ATAC-Seq [22], FAIRE-Seq/MAINE-Seq [30]), approaches that use restriction digestion to fragment DNA (e.g., RAD-Seq [21]), amplicons that vary in length (e.g., fungal ITS sequencing [31]), or techniques such as TAIL-Seq [32] that explicitly seek to measure molecule length. In addition, because the fragmentation pattern of cell-free DNA (cfDNA) is dependent on the chromatin state of the tissue of origin [33], it is also possible that sequencer size bias could influence the measurement of mutant allele fractions in cfDNA [34]. Constructs such as those described here could be routinely spiked into Illumina sequencing runs to monitor size bias, similar to the use of PhiX to report on sequencing error rates and other base-calling metrics.

## Conclusions

We demonstrated that REcount-based measurements of defined plasmid pools are more accurate than PCR-based measurements, that replicate measurements have high precision, and that the technique is amenable to multiplexing through the use of orthogonal restriction enzymes. We used REcount to measure size bias across different Illumina sequencers and found that there are considerable differences in the efficiency of clustering due to molecule length among the different Illumina instruments. We identified sample denaturation as a factor that can influence size bias. We showed that while a randomly sheared RNA-Seq library does not exhibit sequencer-specific quantitative bias in gene expression counts, sequencer size bias can influence both the interpretation of results and the economics of sequencing in cases where library fragment distributions are non-random, such as in RAD-Seq and ATAC-Seq. The quantitative measurements of size bias that we present and the synthetic standards we have developed provide tools for monitoring and accounting for size bias in Illumina sequencing.

## Methods

### Synthesis and cloning of REcount plasmids

#### Even and staggered pool plasmids

The plasmids comprising the even and staggered pools were designed to include a portion of the 16S rRNA gene from 1 of 20 different bacterial species, modeled on the Human Microbiome Project mock microbial communities (HM-276D and HM-277D, [35, 36]), with a 3-bp “TCT” sequence tag added at an analogous position in each construct. These constructs also contained an I-SceI site, allowing for linearization of the plasmids, and a REcount construct, consisting of a unique 20-bp DNA barcode, flanked by Illumina adapters and *Mly*I restriction sites, spaced in a manner to precisely liberate the Illumina adapter-containing barcode construct (Supplemental File 1). These constructs were synthesized as DNA tiles by SGI-DNA and assembled into full-length constructs using the BioXP 3200 (SGI-DNA). The assembled DNA fragments were A-tailed using the A-tailing module from NEB, cloned into pCR2.1 using a TOPO TA cloning kit (Thermo Scientific), and transformed into OneShot TOP10 chemically competent *E. coli* (Thermo Scientific). Multiple colonies were selected, DNA was isolated using a Qiagen Miniprep Kit, and sequence-verified clones were identified by Sanger sequencing with the following primers: M13F: GTAAAACGACGGCCAG and M13R: CAGGAAACAGCTATGAC. The 20 sequence-verified plasmids were quantified using a Quant-iT PicoGreen dsDNA assay (Thermo Fisher Scientific), normalized to 50 ng/μl, and pooled at an equal volume to create the original even pool. The re-pooled even pool and staggered pool were made by adjusting the volume pooled based on the initial PCR-free sequencing data of the original even pool.

#### Orthogonal enzyme multiplexing plasmids

Four synthetic gene fragments were synthesized (Integrated DNA Technologies) in the pIDTSmart-Amp plasmid backbone, consisting of an Illumina adapter-containing construct with internal *Pac*I and *Pme*I sites, and flanked by a pair of either *Mly*I, *Bsm*I, *Bts*^α^I, or *Bsr*DI sites. The full constructs were also flanked by a pair of *Sbf*I sites (Supplemental File 2). In order to make a collection of barcode-containing constructs, the plasmid templates were amplified using the following template-specific primers, and a Golden Gate cloning reaction was used to re-generate the circular plasmid: UMGC_350_MlyI_barcode_p5: NNNNGGTCTCTACTTATCCWWNNNWWNNNAGATCGGAAGAGCGTCGTGTAG; UMGC_350_MlyI_barcode_p7: NNNNGGTCTCTAAGTGCAANNNWWNNNWWAGATCGGAAGAGCACACGTCTGAA; UMGC_350_BsmI_barcode_p5: NNNNGGTCTCTGGTTATCCNNSSNNSSNNAGATCGGAAGAGCGTCGTGTAG; UMGC_350_BsmI_barcode_p7: NNNNGGTCTCTCCAAGCAANNSSNNSSNNAGATCGGAAGAGCACACGTCTGAA; UMGC_350_BtsI_barcode_p5: NNNNGGTCTCTGAACATCCNNNWWNNNWWAGATCGGAAGAGCGTCGTGTAG; UMGC_350_BtsI_barcode_p7: NNNNGGTCTCTGTTCGCAANNNWWNNNWWAGATCGGAAGAGCACACGTCTGAA

UMGC_350_BsrDI_barcode_p5: NNNNGGTCTCTATGAATCCNNSSNNSSNNAGATCGGAAGAGCGTCGTGTAG; and UMGC_350_BsrDI_barcode_p7: NNNNGGTCTCTTCATGCAANNSSNNSSNNAGATCGGAAGAGCACACGTCTGAA.

Briefly, PCR reactions were set up using the following recipe: 1 μl plasmid DNA (20 ng/μl), 2.5 μl primer 1 (10 μM), 2.5 μl primer 2 (10 μM), 19 μl water, and 25 μl 2× Q5 master mix (NEB). PCR amplification was carried out using the following cycling conditions: 98 **°**C for 30 s, followed by 30 cycles of 98 **°**C for 20 s, 60 **°**C for 15 s, 72 **°**C for 1.5 min, followed by 72 **°**C for 5 min. Golden Gate reactions [37, 38] were set up using the following recipe: 1 μl barcoding PCR product from above, 2 μl NEB Cutsmart buffer, 2 μl 10 mM ATP (NEB), 12.5 μl nuclease-free water, 0.5 μl *Bsa*I-HF, 1 μl T4 DNA ligase (NEB 400,000 U/ml), 1 μl *Pac*I. Golden Gate reactions were cycled with the following conditions: 10 cycles of 37 **°**C for 5 min, 21 **°**C for 5 min, then 1 cycle 37 **°**C for 10 min, then 1 cycle 80 **°**C for 20 min. Golden Gate reactions were transformed into chemically competent *E. coli* 5-alpha cells (NEB). Colonies were picked and DNA was isolated using a Qiagen Miniprep Kit. Uniquely barcoded constructs were identified by Sanger sequencing with the following primers: UMGC_350-pIDT-Smart-For: CTGAGGCTCGTCCTGAATGATA and UMGC_350-pIDT-Smart-Rev: ACCGATCATACGTATAATGCCGTAA.

The 12 sequence-verified plasmids were quantified using a Quant-iT PicoGreen dsDNA assay (Thermo Fisher Scientific), normalized to 50 ng/μl, and pooled at equal volume to create the orthogonal enzyme multiplexing test pool. Subsequent NGS analysis indicated that some of these clones were mixed isolates, as other barcodes that had not been detected by Sanger sequencing were present in the NGS data sets. Analysis is based on the Sanger-verified barcodes only.

#### Illumina size standard plasmids

Illumina size standards were designed using three different template molecules as backbones for the variable-length fragment; the 16S rRNA gene (16S) from *E. coli*, the *alpha-Tubulin84B* gene (Tubulin) from *D. melanogaster*, and the *glyceraldehyde-3-phosphate dehydrogenase 1* (GAPDH) gene from *D. melanogaster* (Additional file 1: Figure S5). Any naturally occurring *Mly*I sites in these fragments were modified to remove this restriction site. The variable-length size standards represent nested fragments of these three genes with breakpoints chosen to generate specific molecule lengths, with GC contents between 40 and 60% (Fig. 3, Additional file 1: Figure S5). In order to minimize repetitive sequences, different adapters were used for the normalization and variable size standards (Nextera and TruSeq, respectively), and the normalization and size standards were synthesized in opposite orientations in the construct. Both the Illumina adapter-flanked variable and normalization barcode constructs were flanked by *Mly*I restriction sites. The Illumina size standard constructs were synthesized by GenScript in the pUC57 cloning vector (Supplemental File 3). Approximately 4 μg of each lyophilized plasmid was resuspended in 40 μl of EB (Qiagen). Plasmids were quantified using a Quant-iT PicoGreen dsDNA assay (Thermo Fisher Scientific) and normalized to 10 nM to account for the variable sizes of the plasmids, then pooled at an equimolar ratio.

### qPCR validation of ddPCR assays

A set of primers allowing amplification between the construct-specific barcode and the Illumina flow cell adapter, either in the forward orientation (assay 1, where the construct-specific primer was paired with the p7 primer) or reverse orientation (assay 2, where the construct-specific primer was paired with the p5 primer), were designed and synthesized (Integrated DNA Technologies, Supplemental File 4). In order to validate these assays, we performed qPCR amplification of each individual plasmid, the even plasmid pool, and a negative control (water) with each of the 40 primer sets, as well as a p5/p7 positive control (which is expected to amplify all constructs). PCR reactions were set up as follows: 3 μl template DNA (0.05 ng/μl), 1.06 μl nuclease-free water, 0.6 μl 10× Qiagen PCR buffer, 0.24 μl MgCl_2_ (25 mM), 0.3 μl DMSO, 0.048 μl dNTPs (25 mM), 0.12 μl ROX (25 μM), 0.003 μl SYBR (1000×), 0.03 μl Qiagen Taq (5 U/μl), 0.3 μl primer 1 (10 μM), and 0.3 μl primer 2 (10 μM). Reactions were amplified on an ABI 7900 with the following cycling conditions: 95 °C for 5 min, followed by 35 cycles of 94 °C for 30 s, 55 °C for 30 s, and 72 °C for 30 s, followed by incubation at 72 °C for 1 min. For each primer set, Ct values were normalized to the mean Ct for that primer set across all plasmids and plotted as a heatmap (Additional file 1: Figure S5).

### ddPCR

The re-pooled even plasmid mix was quantified using a Quant-iT PicoGreen dsDNA assay (Thermo Fisher Scientific), diluted to 1 ng/μl, and further diluted 1:10,000 to bring the pool to the correct concentration for digital quantification. The following ddPCR reactions were prepared: 5 μl template DNA, 0.44 μl primer 1 (10 μM), 0.44 μl primer 2 (10 μM), 5.12 μl water, and 11 μl EvaGreen reaction mix (Bio-Rad). In addition, 2 μl of *I-SceI* was added to the ddPCR master mix to linearize the plasmid DNA templates, resulting in between 0.02 and 0.075 μl of *I-SceI* per reaction. Emulsion droplets were generated using a QX200 Droplet Generator (Bio-Rad) following the manufacturer’s instructions, transferred to a 96-well PCR plate, and cycled using the following conditions: 95 °C for 10 min, followed by 40 cycles of 95 °C for 30 s and 55 °C for 1 min, followed by a final extension step of 72 °C for 5 min, and a 12 °C hold. Droplets were counted using a QX200 Droplet Reader (Bio-Rad). The re-pooled even plasmid mix was run in triplicate for both the forward and reverse assays. Single replicates of both the original even pool and the staggered pool were run for both assays. For the staggered pool, the extent of dilution of the 1 ng/μl plasmid pool was varied such that the template abundance of the plasmid targeted by the primer set was expected to be at the correct concentration for digital quantification. Data was analyzed using QuantaSoft Analysis Pro software (BIO-RAD). Replicate measurements were averaged (when available) for both ddPCR assays in order to arrive at a measurement of average ddPCR counts for each construct. Data from the assay was not included in cases where there was no clear separation between positive and negative droplets.

### Sequencing library preparation

#### Even and staggered pool REcount measurements

The following *Mly*I digests were set up for PCR-free quantification: 200–500 ng even or staggered pool DNA, 2 μl Cutsmart buffer (NEB), 1 μl MlyI (NEB), and volume was adjusted to 20 μl with nuclease-free water. Digests were incubated at 37 °C for 1 h, followed by 20 min at 65 °C. Thirty microliters of water was added to each digest (to bring the volume up to 50 μl). Thirty microliters (0.6×) of AmpureXP beads (Beckman Coulter) was added, and after a 5-min incubation, beads were collected on a magnet and the supernatant was transferred to a new tube (discarded beads). Eighty microliters (1×) of AmpureXP beads was added, the beads were washed two times for 30 s using fresh 80% ethanol, and the beads were air dried for 10 min, followed by elution in 20 μl of EB (Qiagen). Libraries were quantified using a Quant-iT PicoGreen dsDNA assay (Thermo Fisher Scientific), fragment sizes were assessed using an Agilent Bioanalyzer High Sensitivity assay, and libraries were normalized to 2 nM for sequencing.

#### Even and staggered pool PCR-based measurements

##### Barcode construct (BC) library preparation

The following PCR reactions were set up to amplify the BC constructs: 1 μl DNA (1 ng/μl), 5 μl 10× Qiagen PCR buffer, 2 μl MgCl_2_ (25 mM), 2.5 μl DMSO, 0.4 μl dNTPs (25 mM), 0.25 μl Qiagen Taq (5 U/μl), 2.5 μl primer 1 (10 μM), 2.5 μl primer 2 (10 μM), and 33.85 μl nuclease-free water.

The following primers were used to amplify the BC constructs: p5: AATGATACGGCGACCACCGA and p7: CAAGCAGAAGACGGCATACGA.

Samples were amplified using the following cycling conditions: 95 °C for 5 min, followed by 10, 20, 30, or 40 cycles of 94 °C for 30 s, 55 °C for 30 s, and 72 °C for 30 s, followed by incubation at 72 °C for 10 min. Libraries were quantified using a Quant-iT PicoGreen dsDNA assay (Thermo Fisher Scientific), fragment sizes were assessed using an Agilent Bioanalyzer High Sensitivity assay, and libraries were normalized to 2 nM for sequencing.

##### V4 fragment library preparation

The following PCR reactions were set up in triplicate to amplify the V4 constructs: 2 μl DNA (0.1 ng/μl), 0.5 μl primer 1 (10 μM), 0.5 μl primer 2 (10 μM), 2 μl nuclease-free water, and 5 μl 2× Q5 master mix. The following primers were used: V4_515F_Nextera: TCGTCGGCAGCGTCAGATGTGTATAAGAGACAGGTGCCAGCMGCCGCGGTAA and V4_806R_Nextera: GTCTCGTGGGCTCGGAGATGTGTATAAGAGACAGGGACTACHVGGGTWTCTAAT.

Reactions were amplified using the following cycling conditions: 98 **°**C for 30 s, followed by 10, 20, 30, or 40 cycles of 98 **°**C for 20 s, 55 **°**C for 15 s, 72 **°**C for 1 min, followed by 72 **°**C for 5 min.

After initial amplification, PCR reactions were diluted 1:60 in nuclease-free water and used as templates in the following indexing reactions: 3 μl PCR 1 (1:60 dilution), 1 μl indexing primer 1 (5 μM), 1 μl indexing primer 2 (5 μM), and 5 μl 2× Q5 master mix. The following indexing primers were used (X indicates the positions of the 8-bp indices): forward indexing primer: AATGATACGGCGACCACCGAGATCTACACXXXXXXXXTCGTCGGCAGCGTC and reverse indexing primer: CAAGCAGAAGACGGCATACGAGATXXXXXXXXGTCTCGTGGGCTCGG.

Reactions were amplified using the following cycling conditions: 98 **°**C for 30 s, followed by 10 cycles of 98 **°**C for 20 s, 55 **°**C for 15 s, 72 **°**C for 1 min, followed by 72 **°**C for 5 min. The full indexing PCR reactions were then purified and normalized using a SequalPrep normalization plate (Thermo Fisher Scientific), followed by elution in 20 μl of elution buffer. An even volume of the normalized libraries was pooled and concentrated using 1× AmpureXP beads (Beckman Coulter). Pooled libraries were quantified using a Qubit dsDNA broad-range assay (Thermo Fisher Scientific), fragment sizes were assessed using an Agilent Bioanalyzer High Sensitivity assay, and libraries were normalized to 2 nM for sequencing.

#### Orthogonal enzyme multiplexing tests

The 12-plasmid orthogonal enzyme pool was cut with 1 of 5 different enzymes (in separate reactions) using the following recipe and enzyme-specific incubation conditions: 20 μl DNA (1 μg), 4 μl NEB buffer (CutSmart or NEB 2.1, depending on enzyme), 2 μl enzyme (either *Mly*I [37 **°**C for 1 h, followed by 65 **°**C for 20 min], *Bsm*I [65 **°**C for 1 h, followed by 80 **°**C for 20 min], *Bts*^α^I [55 **°**C for 1 h], or *Bsr*DI [65 **°**C for 1 h, followed by 80 **°**C for 20 min], or *Sbf*I [37 **°**C for 1 h, followed by 80 **°**C for 20 min]), and 14 μl water. Ten microliters (0.5×) of AmpureXP beads (Beckman Coulter) was added to 20 μl of digested DNA, and after a 5-min incubation, the beads were collected on a magnet and the supernatant was transferred to new tube (discarded beads). Ten microliters of AmpureXP beads was added, and the beads were washed two times for 30 s using fresh 80% ethanol, then air dried for 10 min, before eluting in 20 μl of EB (Qiagen). Libraries were quantified using a Quant-iT PicoGreen dsDNA assay (Thermo Fisher Scientific), fragment sizes were assessed using an Agilent Bioanalyzer High Sensitivity assay, and libraries were normalized to 2 nM for sequencing.

#### Illumina size standards

The following digest of the Illumina size standard pool was set up: 175 μl DNA (10 nM), 20 μl CutSmart buffer (NEB), 5 μl *Mly*I (NEB). The reaction was incubated at 37 **°**C for 1 h, followed by 65 **°**C for 20 min. The library was quantified using a Quant-iT PicoGreen dsDNA assay (Thermo Fisher Scientific), fragment sizes were assessed using an Agilent Bioanalyzer High Sensitivity assay, and libraries were normalized to 2 nM for sequencing.

### RNA-Seq library preparation

Universal Human Reference RNA (Agilent, Catalog number: 740000–41) was processed using a Truseq Stranded mRNA Sample Preparation Kit (Illumina). Briefly, 1 μg of total RNA was oligo-dT purified using oligo-dT-coated magnetic beads, fragmented, and then reverse transcribed into cDNA. The cDNA was adenylated and then ligated to dual-indexed (barcoded) adaptors using TruSeq RNA CD Indices (Illumina) and amplified using 15 cycles of PCR according to the Truseq Stranded mRNA Sample Preparation Kit protocol. The library was quantified using a Quant-iT PicoGreen dsDNA assay (Thermo Fisher Scientific), fragment sizes were assessed using an Agilent Bioanalyzer High Sensitivity assay, and libraries were normalized to 2 nM for sequencing.

### RAD-Seq library preparation

DNA was extracted from *Drosophila melanogaster* strains using the Insect Supplementary Protocol for the DNeasy Blood and Tissue Kit (Qiagen). The following strains were tested:

**Table:** 

Stock name	Source	Notes
Berlin-K	Bloomington Drosophila Stock Center	RRID:BDSC_8522
Canton-S	Bloomington Drosophila Stock Center	RRID:BDSC_64349
DGRP-21	Bloomington Drosophila Stock Center	RRID:BDSC_28122
DGRP-26	Bloomington Drosophila Stock Center	RRID:BDSC_28123
DGRP-48	Bloomington Drosophila Stock Center	RRID:BDSC_55016
DGRP-100	Bloomington Drosophila Stock Center	RRID:BDSC_55017
Genome Strain	Bloomington Drosophila Stock Center	RRID:BDSC_2057
IsoD1	Clandinin Lab, Stanford University	[39]
Ore-R-C	Bloomington Drosophila Stock Center	RRID:BDSC_5
Ore-R-modENCODE	Bloomington Drosophila Stock Center	RRID:BDSC_25211

One hundred nanograms of genomic DNA was digested at 37 °C for 2 h with PstI-HF (10 U, NEB; New England Biolabs) in Cutsmart buffer. Digested DNA was ligated to TGCA-overhang adaptors at a final concentration of 0.1 μM for each adaptor (Integrated DNA Technologies). Nextera-style adapters containing 0–6 frameshifting bases downstream of the sequencing primer binding site were incubated at 22 °C for 1 h followed by heat inactivation of T4 ligase (400 U, NEB) at 65 °C for 20 min. After a SPRI cleanup, half the volume of the adapter-ligated DNA fragments was amplified using NEBNext High-Fidelity 2× PCR Master Mix Taq (NEB) with Forward and Reverse indexing primers (see above) at a final concentration of .5 μM for each primer using the following cycling conditions: initial denaturation at 98 °C for 30 s followed by 18 cycles of 98 °C for 10 s, 55 °C for 30 s, and 72 °C for 30 s with a final extension step at 72 °C for 5 min. Purified libraries were quantified with the Quant-IT PicoGreen dsDNA assay (Thermo Fisher Scientific) and pooled by mass, and adaptor dimers were removed using a 1× SPRI bead purification. The final library fragment size distribution was assessed using an Agilent Bioanalyzer High Sensitivity assay, and libraries were normalized to 2 nM for sequencing.

### ATAC-Seq library preparation

ATAC-Seq was performed following the protocol described by Buenrostro and colleagues [40]. Propagation and embryoid body (EB)-based differentiation of the doxycycline-inducible *Pax3* ES cell line was performed as previously described [41]. *Pax3* induction was achieved by adding doxycycline (final concentration of 1 μg/ml) in 3-day EB cultures. Fifty thousand freshly sorted PDGFRa+FLK1− cells from cultures differentiated for 4 days (non-induced and 1-day Pax3-induced cells) were washed with 200 μl of cold PBS then resuspended in 100 μl of cold lysis buffer (10 mM Tris-HCl pH 7.4, 10 mM NaCl, 3 mM MgCl_2_, 0.1% IGEPAL CA-630), spun at 500 g for 10 min at 4 °C, and resuspended in 50 μl of the transposition reaction mix. Transposition occurred at 37 °C for 30 min, after which transposed DNA was purified using a Qiagen MinElute Kit and eluted in 12 μl Elution Buffer. Transposed DNA was quantified using qPCR, followed by the final PCR amplification using Illumina-compatible adapter-barcodes (using the forward indexing primers and reverse indexing primers described above). Three independent libraries were generated for both non-induced and *Pax3*-induced cells. Libraries were quantified using a Qubit dsDNA broad-range assay (Thermo Fisher Scientific), fragment sizes were assessed using an Agilent Bioanalyzer High Sensitivity assay, and libraries were normalized to 2 nM for sequencing.

### Sequencing

DNA libraries were denatured with NaOH and prepared for sequencing according to the protocols described in the Illumina iSeq, MiSeq, NextSeq, HiSeq 2500, HiSeq 4000, and NovaSeq Denature and Dilute Libraries Guides. Libraries were generally sequenced along with other samples in a fraction of a sequencing lane.

### Data analysis

#### REcount data analysis

Demultiplexed fastq files were generated using Illumina’s bcl2fastq software. REcount data was analyzed using custom R and Python scripts and BioPython [42]. The first 20 bp of the sequencing reads was mapped against a barcode reference file (Supplemental Files 5–8), with a maximum of two mismatches allowed, using a custom script which is available on GitHub (https://github.com/darylgohl/REcount). Additionally, test data and expected output files are also available at https://github.com/darylgohl/REcount/tree/master/REcount_test_data. Analysis of the V4 amplicon data was performed using the reference-based mapping pipeline described here: https://bitbucket.org/jgarbe/gopher-pipelines/wiki/metagenomics-pipeline.rst, using the reference file in Supplemental File 9 to build the bowtie2 index [43]. For the analysis of quality scores (Additional file 1: Figure S5), the data for all runs on a given platform was concatenated into a single fastq file, then split into individual fastq files for each individual construct, based on the 20-bp sequence barcodes in each construct. Next, the reads were trimmed to 50 bp using cutadapt [44], so that all constructs and sequencing runs could be compared in a standardized manner. Mean quality scores were calculated for each construct that was represented by at least 100 reads in the data set. This analysis was carried out using a custom Python script (Supplemental File: REcount_split_fastq_Q-score_plots.py), which is available at: https://github.com/darylgohl/REcount/tree/master/Q-score_Analysis.

#### RNA-Seq data analysis

Data quality was assessed using FastQC (http://www.bioinformatics.babraham.ac.uk/projects/fastqc/). Low-quality bases and adapter sequences were removed using Trimmomatic [45]. Reads were aligned to the human genome assembly GRCh38 using Hisat2 [46]. FPKM expression values were generated using Cuffquant and Cufnorm from the Cufflinks package [47], and Raw read counts were generated using featureCounts from the Subread R package [48].

#### RAD-Seq data analysis

Demultiplexed fastq files were generated using Illumina’s bcl2fastq software. Fastq files with more than 500,000 reads were subsampled down to 500,000 reads. The first eight bases were removed from the beginning of each read in order to remove adapter sequences, and Trimmomatic [45] was used to remove adapter sequences at the 3′ ends of reads. The paired-end fastq files were aligned to the *Drosophila melanogaster* r6.08 reference genome using bwa [49]. Freebayes [50] was used to jointly call variants across all samples simultaneously. The raw VCF file generated by Freebayes was filtered using VCFtools [51] to remove variants with minor allele frequency < 1%, variants with genotype rates < 95%, and samples with genotype rates < 50%.

#### ATAC-Seq data analysis

Demultiplexed fastq files were generated using Illumina bcl2fastq software. Paired-end reads were mapped to the mouse genome (mm10) using bowtie2 [43]. The resulting SAM files were filtered to remove mitochondrial reads, and SAMtools [52] was used to remove duplicate reads. Custom Python scripts were used to subsample the SAM files to a depth of 20 million reads (Supplemental File: ATAC_Seq_Sam_Subsampler.py) and to split the subsampled SAM files into non-nucleosomal and nucleosomal bins (Supplemental File: ATAC_Seq_Split_Nucleosomes.py), using the following size ranges specified by Buenrostro et al. [22]: non-nucleosomal: < 100 bp; mono-nucleosomal: 180–247 bp; di-nucleosomal: 315–473 bp; and tri-nucleosomal: 558–615 bp. MACS [28] was used to call peaks that were induced by PAX3 expression, and IGV [53] was used to visualize read pileups and MACS peaks. Peaks detected in two out of three samples were identified using BEDTools [54].

## Additional file

Additional file 1: **Figure S1.** Initial and re-pooled even plasmid pool data. Figure S2. Lack of correlation between BC and V4 PCR. Figure S3. Droplet digital PCR assay validation and data. Figure S4. Assessment of REcount measurements of a staggered plasmid pool. Figure S5. Illumina size standard pool composition and data. Figure S6 Context-specific effects on clustering of size standards. Figure S7. Size distribution of pooled RAD-Seq library. Figure S8. Insert size distribution, missing genotype calls, and mean read depth for RAD-Seq samples. Figure S9. Insert size distributions of individual ATAC-Seq libraries. Figure S10 Distribution of mapped reads at the *Fgfr4* locus at different subsampling depths. (PDF 1657 kb)
