# Supplementary material for: Measuring sequencer size bias using REcount: a novel method for highly accurate Illumina sequencing-based quantification
Source: Genome Biol. 2019 Apr 29;20:85. doi: 10.1186/s13059-019-1691-6 (PMC6489363; doi:10.1186/s13059-019-1691-6)
Supplement: Supplementary file 1 — Figure S1. Initial and re-pooled even plasmid pool data. Figure S2. Lack of correlation between BC and V4 PCR. Figure S3. Droplet digital PCR assay validation and data. Figure S4. Assessment of REcount measurements of a staggered plasmid pool. Figure S5. Illumina size standard pool composition and data. Figure S6 Context-specific effects on clustering of size standards. Figure S7. Size distribution of pooled RAD-Seq library. Figure S8. Insert size distribution, missing genotype calls, and mean read depth for RAD-Seq samples. Figure S9. Insert size distributions of individual ATAC-Seq libraries. Figure S10 Distribution of mapped reads at the Fgfr4 locus at different subsampling depths. (PDF 1657 kb) [file 13059_2019_1691_MOESM1_ESM.pdf]

Supplemental Data Files for:

Measuring sequencer size bias using REcount: a novel method for highly accurate Illumina sequencing-based quantification

I. Supplemental Figures

Fig. S1
Initial and re-pooled even plasmid pool data.

Fig. S2
Lack of correlation between BC and V4 PCR.

Fig. S3
Droplet digital PCR assay validation and data.

Fig. S4
Assessment of REcount measurements of a staggered plasmid pool.

Fig. S5
Illumina size standard pool composition and data.

Fig. S6
Context-specific effects on clustering of size standards.

Fig. S7
Size distribution of pooled RAD-Seq library.

Fig. S8
Insert size distribution, missing genotype calls, and mean read depth for RAD-Seq samples.

Fig. S9
Insert size distributions of individual ATAC-Seq libraries.

Fig. S10
Distribution of mapped reads at the *Fgfr4* locus at different subsampling depths.

II. Supplemental Files

(Available at: https://github.com/darylgohl/REcount/tree/master/Supplemental_Files)

1. Supplemental_File_1.fasta
   Sequences of the synthetic DNA standards used to construct the even and staggered plasmid pools.

2. Supplemental_File_2.fasta
   Sequences of the synthetic DNA used to construct the orthogonal restriction enzyme plasmids.

3. Supplemental_File_3.fasta
   Sequences of the synthetic DNA inserts from the Illumina size standard plasmids.

4. Supplemental_File_4.xlsx

A table of the primers used for the ddPCR assays in this study.

5. Supplemental_File_5.fasta
   REcount barcode mapping file from the even and staggered plasmid pools.

6. Supplemental_File_6.fasta
   Expected REcount barcodes for orthogonal enzyme multiplexing tests.

7. Supplemental_File_7.fasta
   Normalization barcode mapping file for Illumina size standards.

8. Supplemental_File_8.fasta
   Variable barcode mapping file for Illumina size standards.

9. Supplemental_File_9.fasta
   Reference sequences for V4 PCR mapping.

10. REcount_split_fastq_Q-score_plots.py
    Script for analyzing Q-scores of synthetic size standards.

11. ATAC_Seq_Sam_Subsampler.py
    Python script for used for subsampling SAM flies for ATAC-Seq analysis.

12. ATAC_Seq_Split_Nucleosomes.py
    Python script for used for splitting SAM flies into non-nucleosomal and nucleosomal bins for ATAC-Seq analysis.

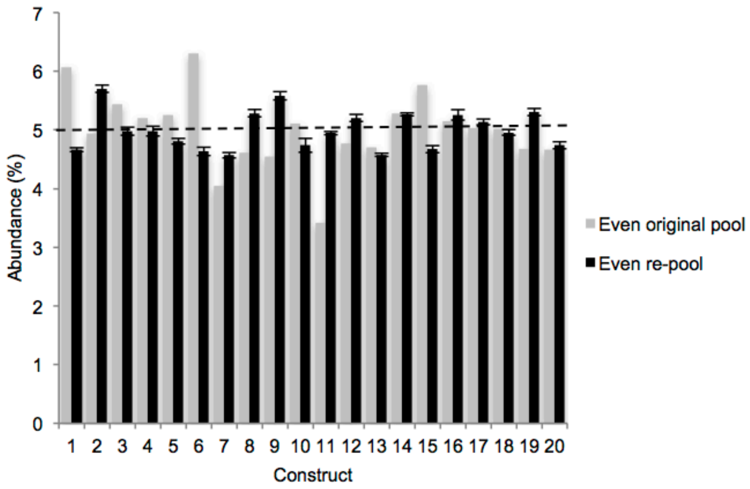

Fig. S1. Initial and re-pooled even plasmid pool data. REcount measurements of an initial attempt at even plasmid pooling based on PicoGreen data, and a subsequent re-pooling informed by the initial pool sequencing data.

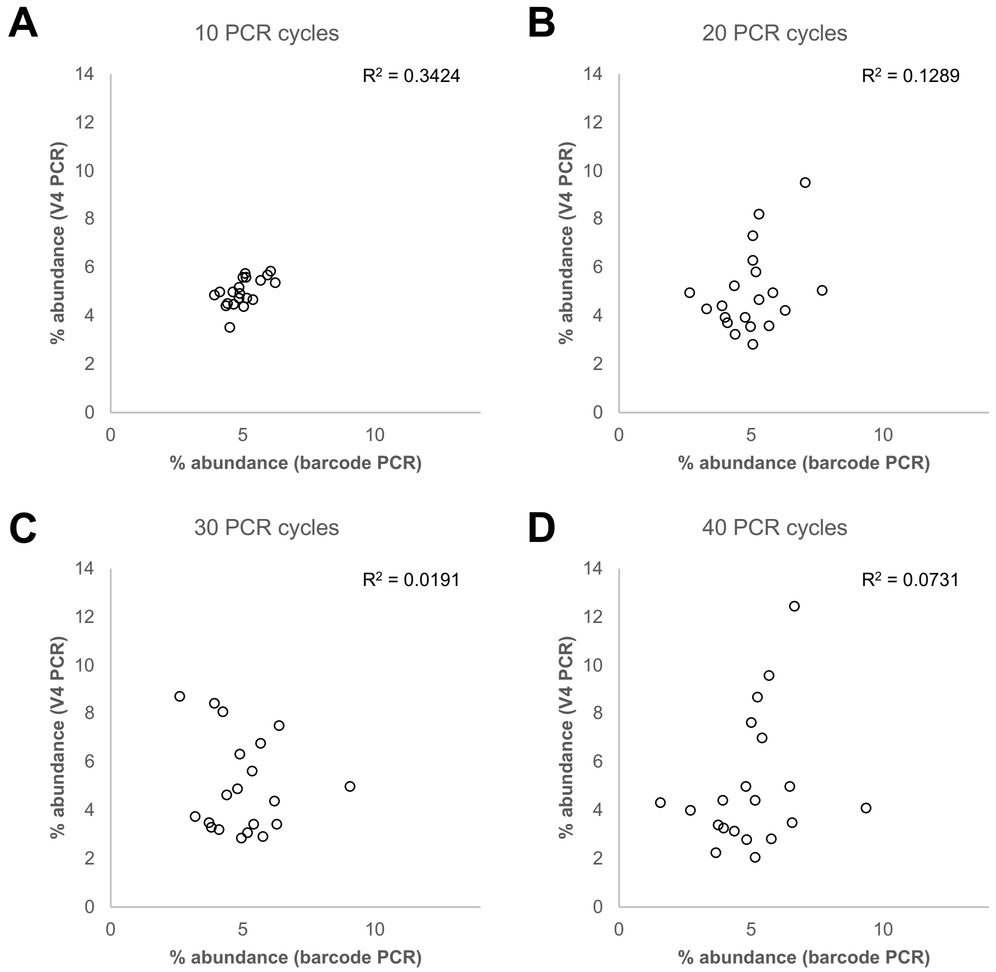

Fig. S2. Lack of correlation between BC and V4 PCR. Scatterplots of BC and V4 abundance data for the even plasmid pool, when amplified for A) 10, B) 20, C) 30, or D) 40 PCR cycles.

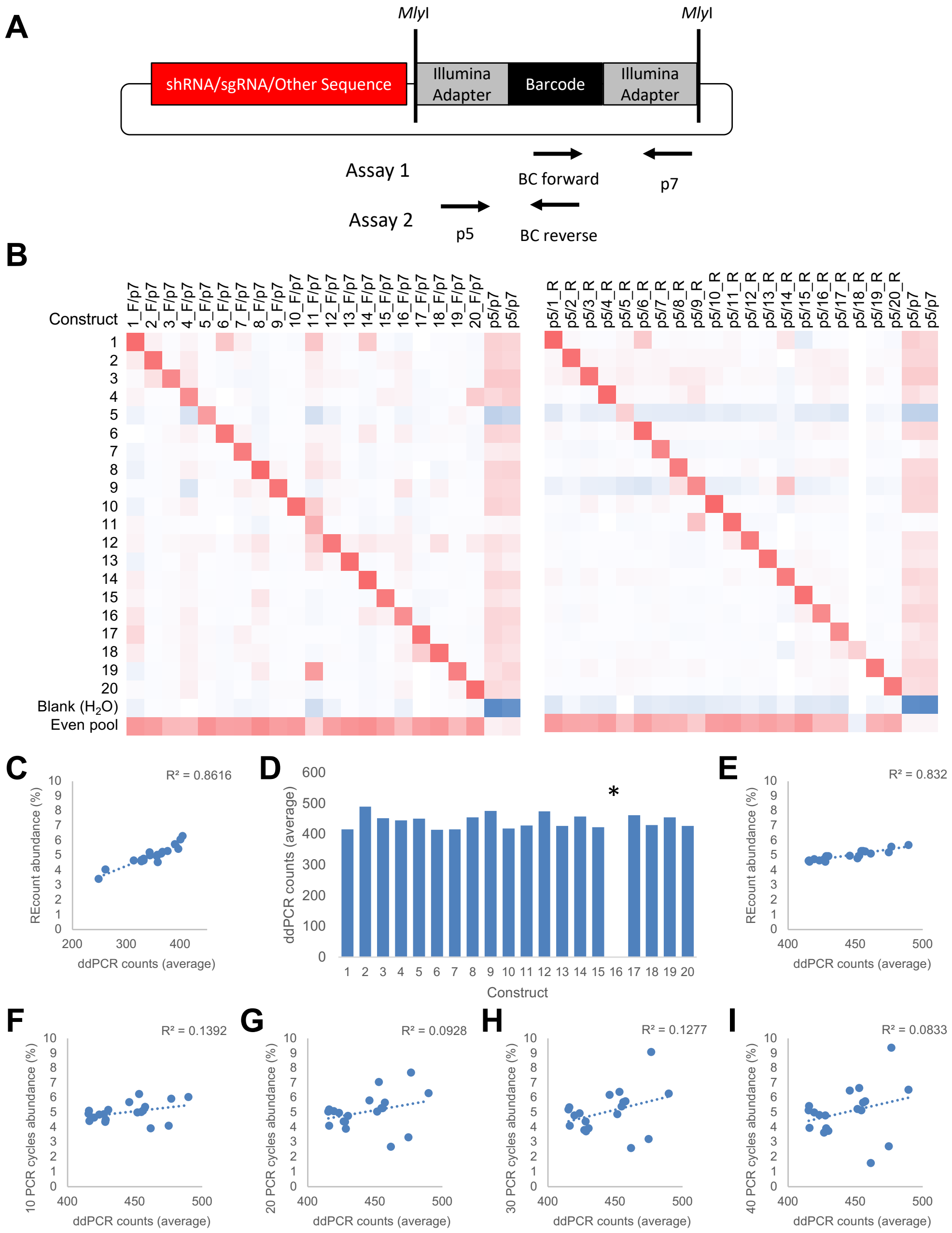

Fig. S3. Droplet digital PCR assay validation and data. A) Schematic depicting the two ddPCR assays that were developed for each construct in the plasmid pool. B) qPCR data showing the specificity of each assay for the target construct as assessed by amplification of each individual plasmid, the even plasmid pool, or a negative control, with each primer pair. C) Correlation between ddPCR data and REcount quantification for the original even plasmid pool. D) ddPCR counts for the re-pooled even plasmid pool. Bars are the average of triplicates of the forward and reverse ddPCR assays where data could be generated for both assays, or just the forward or reverse assay in the case where one assay failed. *For plasmid 16, both the forward and reverse assays failed and thus no ddPCR information is available for this construct. E) Correlation between ddPCR data and REcount quantification for the re-pooled even plasmid pool. F-I) Correlation between ddPCR data and BC PCR-based quantification of the re-pooled even plasmid pool amplified for F) 10, G) 20, H) 30, I) 40 PCR cycles.

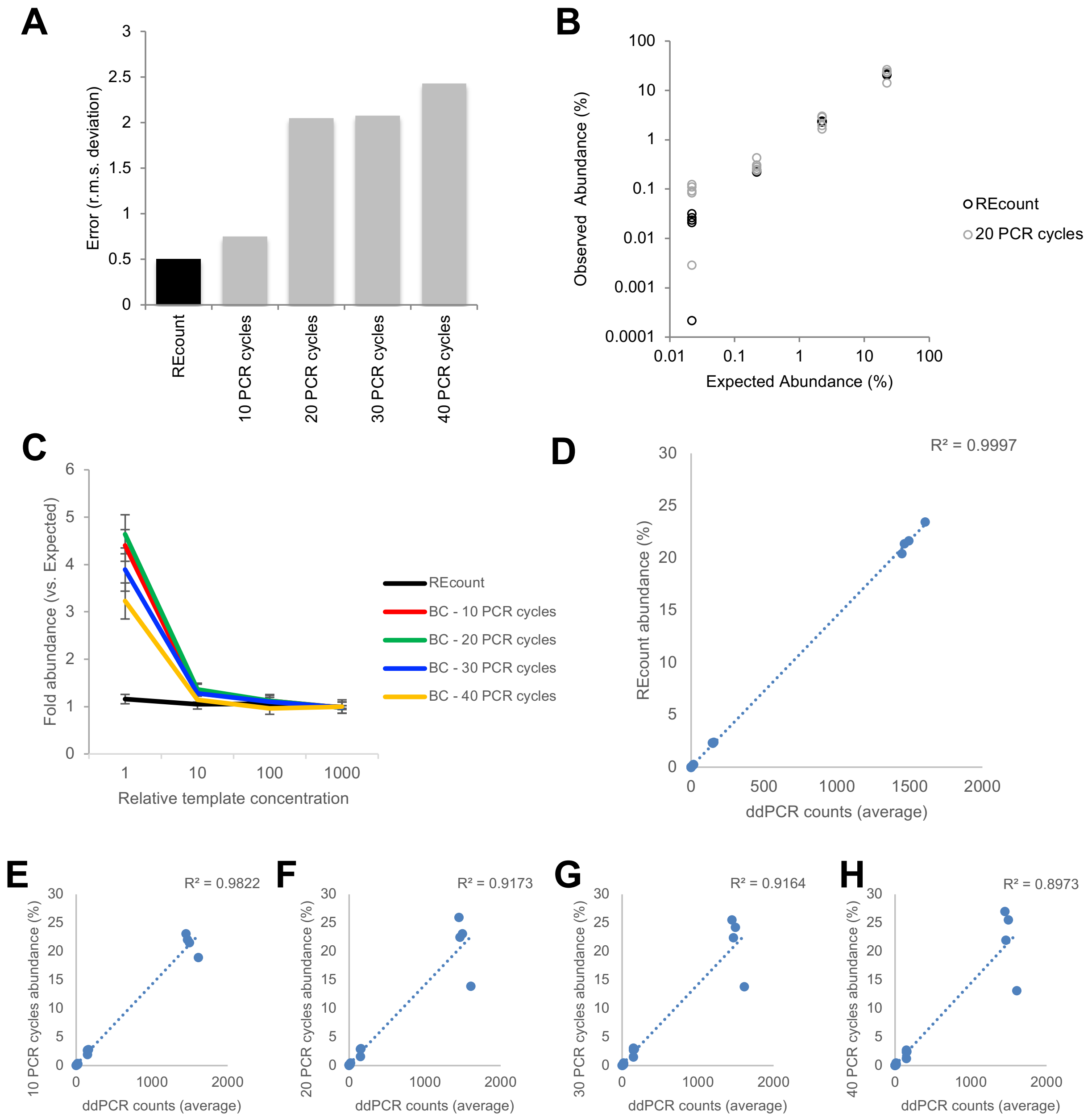

Fig. S4. Assessment of REcount measurements of a staggered plasmid pool. A) Root mean squared deviation from expected values when the staggered plasmid pool is measured using REcount, and varying cycles of PCR amplification of the barcode construct. B) Comparison of REcount and PCR-based measurements of the staggered plasmid pool. C) Average measured representation of constructs pooled at different levels relative to expected values when measured using REcount or varying cycles of PCR. D) Correlation of ddPCR data and REcount measurements of the staggered plasmid pool. E-H) Correlation between ddPCR data and BC PCR-based quantification of the staggered plasmid pool amplified for E) 10, E) 20, G) 30, H) 40 PCR cycles. Error bars are +/- s.e.m.

A

Construct size	Gene	GC content (%)	Construct size	Gene	GC content (%)	Construct size	Gene	GC content (%)
150	16S rRNA (E. coli)	45.45	150	GAPDH (D. melanogaster)	45.45	150	Tubulin (D. melanogaster)	54.55
300	16S rRNA (E. coli)	51.16	300	GAPDH (D. melanogaster)	43.02	300	Tubulin (D. melanogaster)	43.60
450	16S rRNA (E. coli)	53.73	450	GAPDH (D. melanogaster)	40.99	450	Tubulin (D. melanogaster)	50.00
600	16S rRNA (E. coli)	54.87	600	GAPDH (D. melanogaster)	47.25	600	Tubulin (D. melanogaster)	52.75
750	16S rRNA (E. coli)	53.70	750	GAPDH (D. melanogaster)	50.16	750	Tubulin (D. melanogaster)	55.79
900	16S rRNA (E. coli)	54.79	900	GAPDH (D. melanogaster)	52.59	900	Tubulin (D. melanogaster)	56.87
1050	16S rRNA (E. coli)	55.31	1050	GAPDH (D. melanogaster)	55.31	1050	Tubulin (D. melanogaster)	56.29
1200	16S rRNA (E. coli)	54.94	1200	GAPDH (D. melanogaster)	55.50	1200	Tubulin (D. melanogaster)	56.72
1350	16S rRNA (E. coli)	54.66	1350	GAPDH (D. melanogaster)	55.65	1350	Tubulin (D. melanogaster)	56.87
1500	16S rRNA (E. coli)	54.52	1500	GAPDH (D. melanogaster)	55.10	1500	Tubulin (D. melanogaster)	57.58

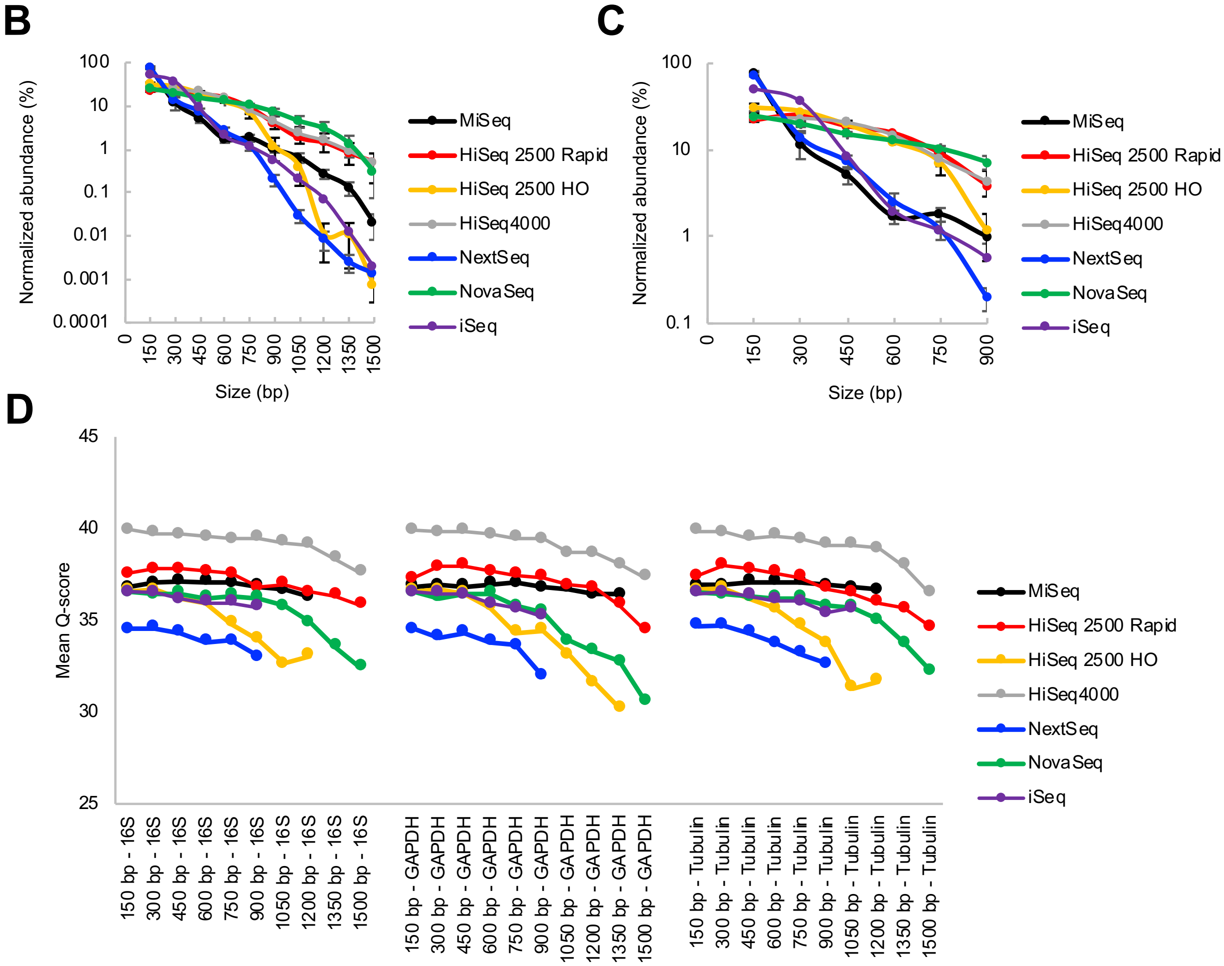

Fig. S5. Illumina size standard pool composition and data. A) Composition of the Illumina size standard constructs, which consist of three different backbone molecules (16S rRNA, GAPDH, and Tubulin), ranging from 150 bp to 1500 bp in length. B) Size bias profiles of the iSeq (n=1 flow cell), MiSeq (n=6 flow cells), HiSeq 2500 Rapid (n=1 flow cell, 2 lanes), HiSeq 2500 High Output (HO, n=2 flow cells, 10 lanes), HiSeq 4000 (n=3 flow cells, 6 lanes), NextSeq (n=4 flow cells), and NovaSeq (n=4 flow cells, 4 lanes) sequencers. C) Size bias profiles restricted to fragment lengths of 900 bp or less. D) Platform and construct-specific mean quality scores for the Illumina size standard constructs for the first 50 bp of read 1.

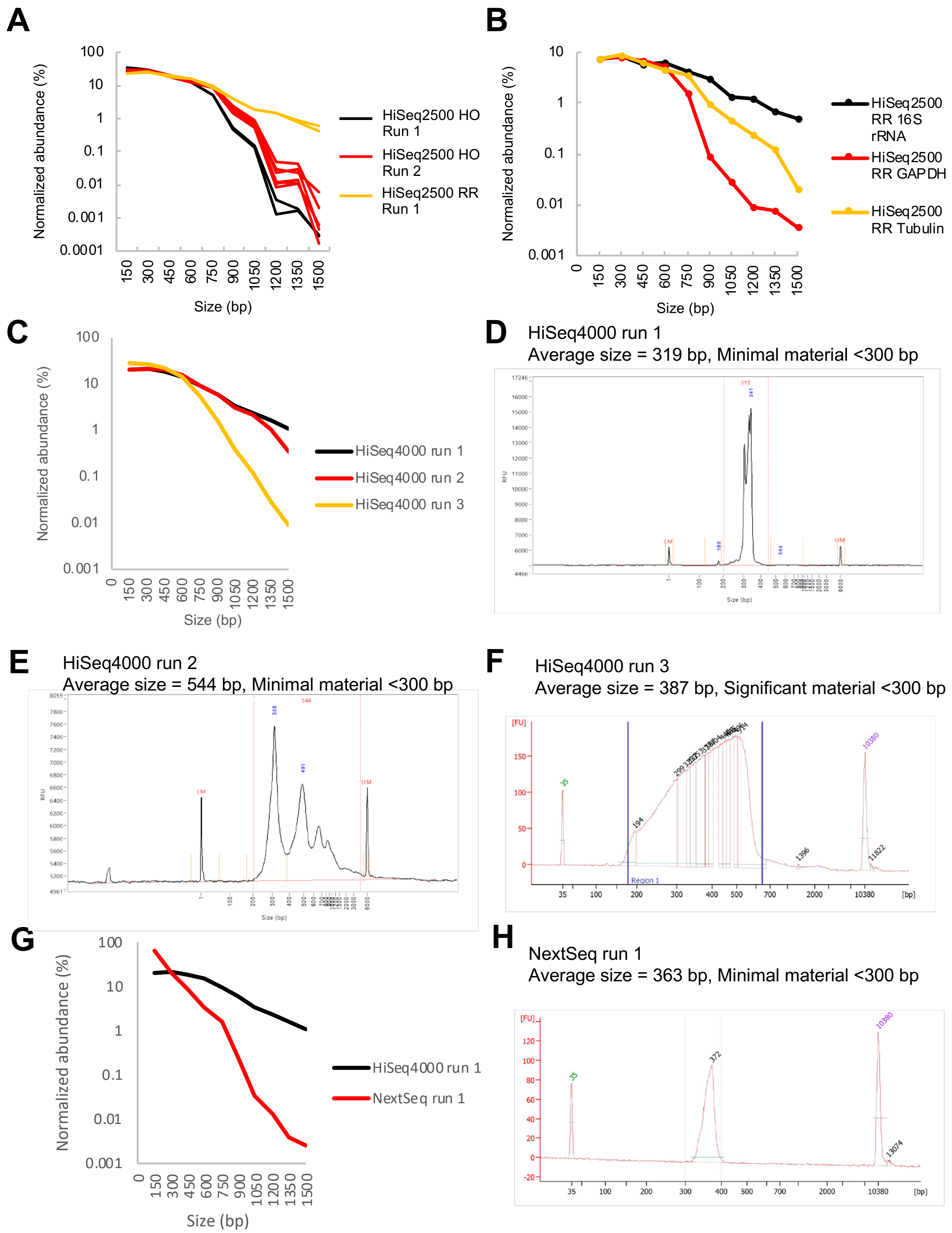

Fig. S6. Context-specific effects on clustering of size standards. A) Between lane and between flow cell differences in size bias profiles for HiSeq2500 Rapid Run (on-board clustering) and HiSeq2500 High Output (cBot clustering). B) Template-specific size biases

observed on the HiSeq2500 in Rapid Run mode. C) Differences in size standard measurements for three HiSeq 4000 runs (3 different flow cells). D) Fragment size profile of the library run together with the size standards in run 1 of the HiSeq 4000. E) Fragment size profile of the library run together with the size standards in run 2 of the HiSeq 4000. F) Fragment size profile of the library run together with the size standards in run 3 of the HiSeq 4000. G) Differences in size standard measurements for run 1 of the HiSeq 4000 and run 1 of the NextSeq. H) Fragment size profile of the library run together with the size standards in run 1 of the NextSeq.

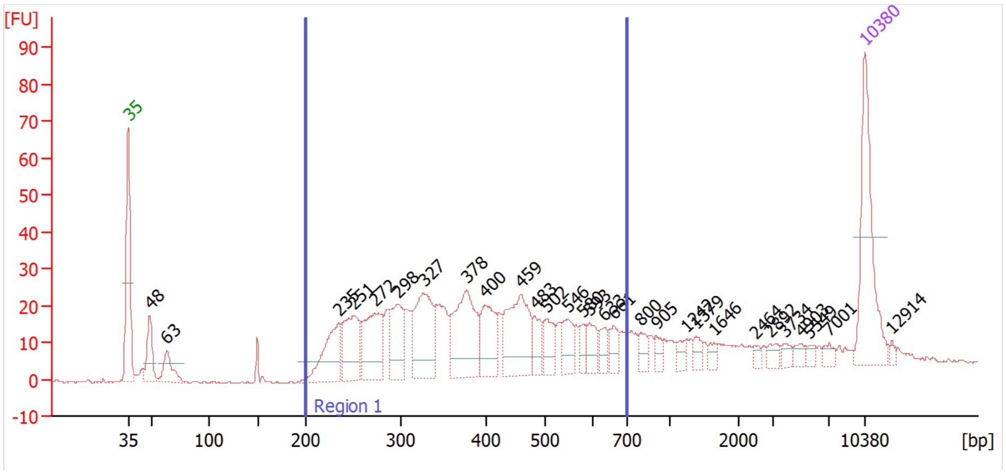

Fig. S7. Size distribution of pooled RAD-Seq library. Agilent Bioanalyzer trace for the 11 pooled RAD-Seq libraries.

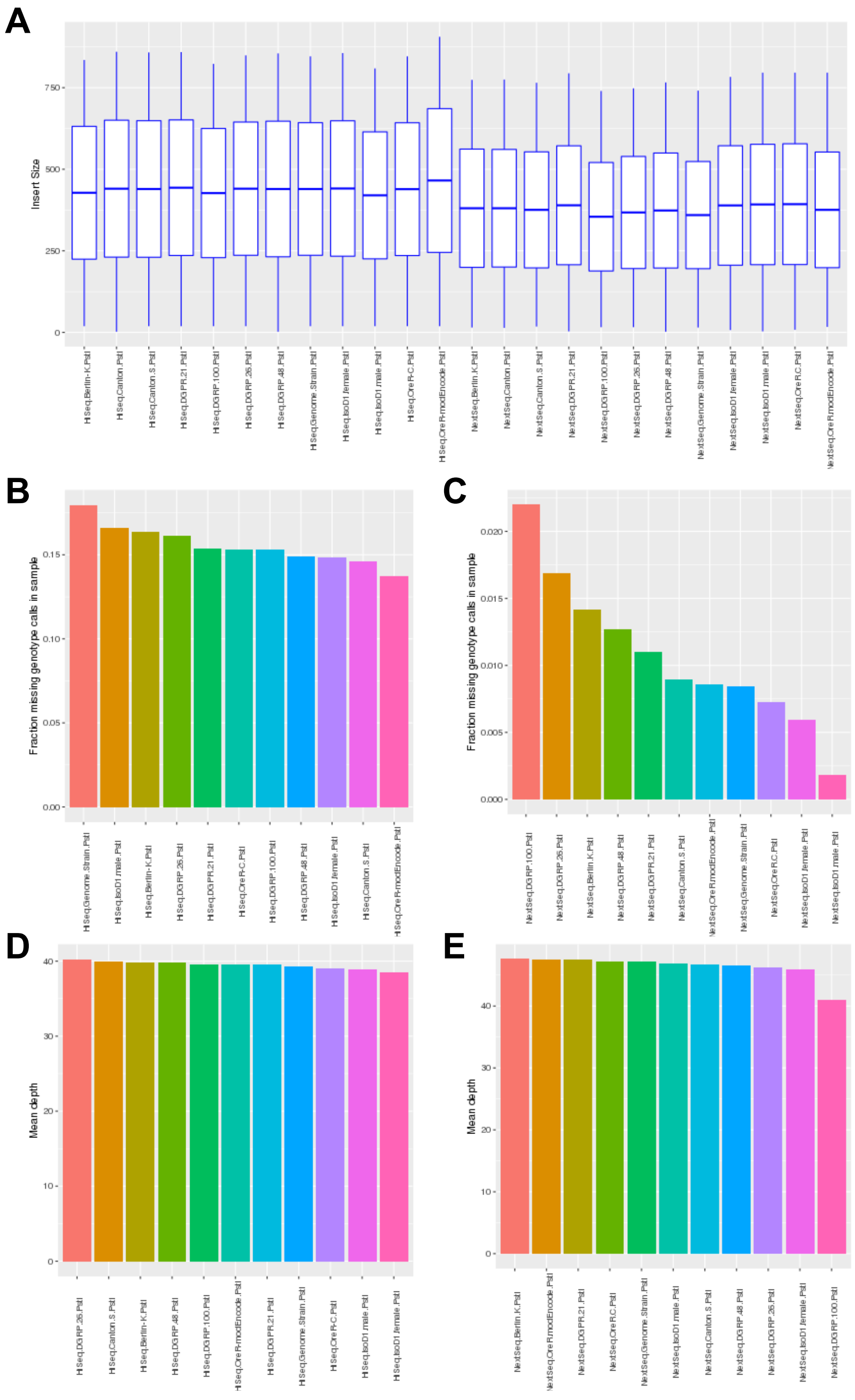

Fig. S8. Insert size distribution, missing genotype calls, and mean read depth for RAD-Seq samples. A) Insert size distributions for the 11 RAD-Seq libraries sequenced on either the HiSeq or the NextSeq. B) Fraction of missing genotype calls for RAD-Seq libraries sequenced on the HiSeq. C) Fraction of missing genotype calls for RAD-Seq libraries sequenced on the NextSeq. D) Mean read depth of markers for RAD-Seq libraries sequenced on the HiSeq. E) Mean read depth of markers for RAD-Seq libraries sequenced on the NextSeq.

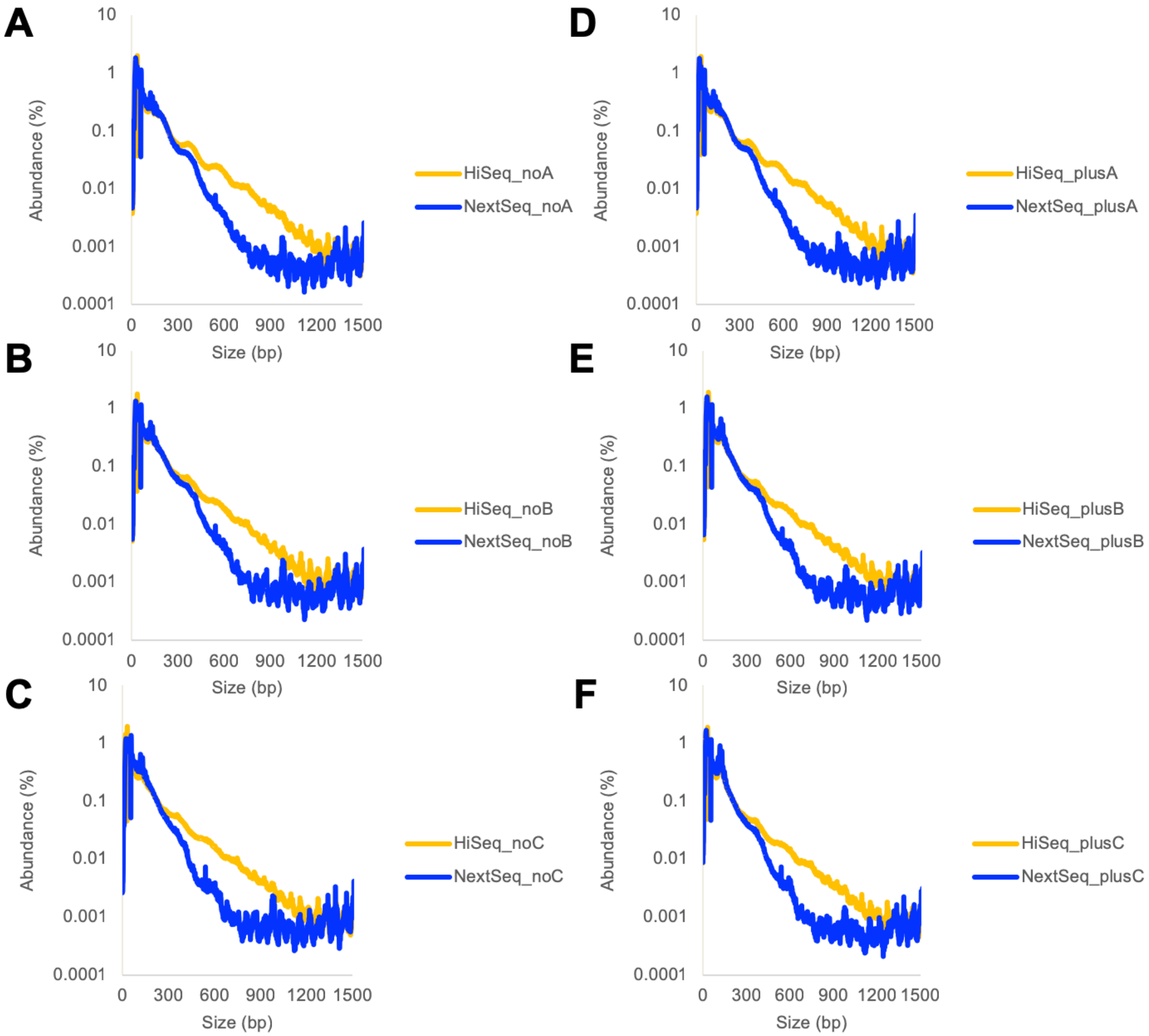

Fig. S9. Insert size distributions of individual ATAC-Seq libraries. A-F) Insert size distributions for the 6 individual ATAC-Seq libraries sequenced on the HiSeq or the NextSeq.

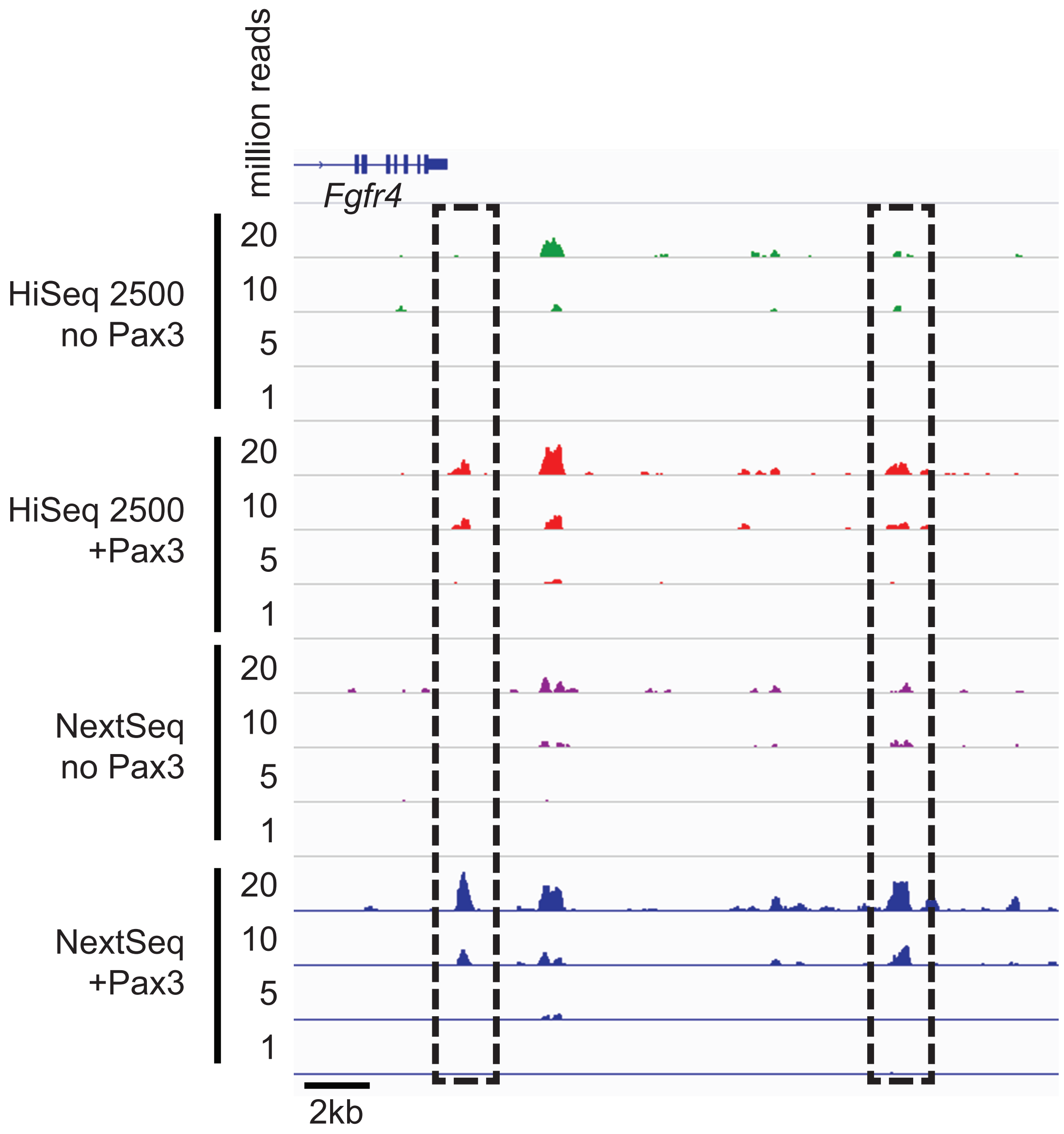

Fig. S10. Distribution of mapped reads at the *Fgfr4* locus at different subsampling depths. Effect of read depth on PAX3-responsive enhancer peaks.
